# Quantitative and qualitative plant-pathogen interactions call upon similar pathogenicity genes with a spectrum of effects

**DOI:** 10.3389/fpls.2023.1128546

**Published:** 2023-05-10

**Authors:** Camilla Langlands-Perry, Anaïs Pitarch, Nicolas Lapalu, Murielle Cuenin, Christophe Bergez, Alicia Noly, Reda Amezrou, Sandrine Gélisse, Célia Barrachina, Hugues Parrinello, Frédéric Suffert, Romain Valade, Thierry C. Marcel

**Affiliations:** ^1^ Université Paris-Saclay, INRAE, UR BIOGER, Palaiseau, France; ^2^ ARVALIS Institut du Végétal, Boigneville, France; ^3^ MGX-Montpellier GenomiX, Univ. Montpellier, CNRS, INSERM, Montpellier, France

**Keywords:** quantitative pathogenicity, quantitative trait loci, small secreted proteins, Septoria tritici blotch (STB), *Triticum aestivum* (L.)

## Abstract

Septoria leaf blotch is a foliar wheat disease controlled by a combination of plant genetic resistances and fungicides use. *R-*gene-based qualitative resistance durability is limited due to gene-for-gene interactions with fungal avirulence (*Avr*) genes. Quantitative resistance is considered more durable but the mechanisms involved are not well documented. We hypothesize that genes involved in quantitative and qualitative plant-pathogen interactions are similar. A bi-parental population of *Zymoseptoria tritici* was inoculated on wheat cultivar ‘Renan’ and a linkage analysis performed to map QTL. Three pathogenicity QTL, *Qzt-I05-1, Qzt-I05-6* and *Qzt-I07-13*, were mapped on chromosomes 1, 6 and 13 in *Z. tritici*, and a candidate pathogenicity gene on chromosome 6 was selected based on its effector-like characteristics. The candidate gene was cloned by *Agrobacterium tumefaciens*-mediated transformation, and a pathology test assessed the effect of the mutant strains on ‘Renan’. This gene was demonstrated to be involved in quantitative pathogenicity. By cloning a newly annotated quantitative-effect gene in *Z. tritici* that is effector-like, we demonstrated that genes underlying pathogenicity QTL can be similar to *Avr* genes. This opens up the previously probed possibility that ‘gene-for-gene’ underlies not only qualitative but also quantitative plant-pathogen interactions in this pathosystem.

## 1 Introduction

Plant-pathogenic microorganisms employ a variety of strategies to successfully infect crops and effectors are very often involved in infection mechanisms (Koeck et al., 2011; Fouché et al., 2018 ; Shao et al., 2021). Effectors are molecules that manipulate host immunity to enable parasitic infection (Kamoun, 2006; Pradhan et al., 2021). They are generally cysteine-rich small secreted proteins (SSP) (Houterman et al., 2009; Stergiopoulos and Wit, 2009; Oliva et al., 2010) and show low similarities with other species (Plissonneau et al., 2017). They are among the most polymorphic genes found in pathogen genomes (Win et al., 2012), often found in highly plastic transposable element-rich regions of the genome (Ma et al., 2010; Soyer et al., 2014; Fouché et al., 2018; Plissonneau et al., 2018).

As an answer to the onslaught brought on by effectors, host-plants have evolved *R* resistance genes that encode proteins capable of recognizing effectors, thus triggering a defensive response (Petit-Houdenot and Fudal, 2017). In this context, effectors are referred to as avirulence (*Avr*) genes, and the *R/Avr* interaction follows a gene-for-gene interaction as defined by Flor (Flor, 1971). Gene-for-gene interactions have been described in many pathosystems (Wit, 1995; Jia et al., 2000; Gout et al., 2006; Hall et al., 2009; Stukenbrock and McDonald, 2009). Almost exclusively associated with qualitative resistance, these interactions pose an issue for disease resistance durability in crops because of the strong selective pressure they impose on pathogen populations. Indeed, a single mutation in the *Avr* gene is sufficient to overcome the disease resistance provided by the *R* gene as the effector will no longer be recognized by the plant’s defence mechanisms (Niks et al., 2015).

Quantitative resistance imposes lower selective pressure on populations as it is polygenic, based on a combination of loci with varying effects all contributing to an overall more or less resistant phenotype (Niks et al., 2015). It is therefore widely thought to be able to slow pathogen adaptation and to be more durable than qualitative resistance. Mechanisms underlying quantitative resistance are not well known but have been hypothesized (Poland et al., 2009). Some hypotheses suggest that gene-for-gene interactions similar to those involved in qualitative resistance are in play, but this has been shown only in very isolated cases (Leonards-Schippers et al., 1994; Qi et al., 1999; Arru et al., 2003; González et al., 2012; Meile et al., 2018; Jiquel et al., 2021).

Septoria tritici blotch (STB), caused by the ascomycete fungus *Zymoseptoria tritici*, is one of the most devastating diseases of wheat in Europe. It is responsible for high yield losses worldwide, 30 to 50% loss when environmental conditions are favourable to the disease’s development (Eyal et al., 1987; Fones and Gurr, 2015). Known sources of resistance in wheat to STB comprise 22 major resistance genes (Brown et al., 2015; Yang et al., 2018) and over 100 resistance quantitative trait loci (QTL) detected genome-wide (Brown et al., 2015; Gerard et al., 2017; Vagndorf et al., 2017; Karlstedt et al., 2019; Yates et al., 2019; Langlands-Perry et al., 2022). Very few genes are known to be involved in pathogenicity for *Z. tritici* (Marshall et al., 2011; Poppe et al., 2015; Rudd et al., 2015; Hartmann et al., 2017; Kettles et al., 2017; Yemelin et al., 2022), and qualitative gene-for-gene interactions have been demonstrated for the *T. aestivum-Z. tritici* pathosystem with the *Stb6*/*AvrStb6* and *Stb9*/*AvrStb9* interactions (Brading et al., 2002; Zhong et al., 2017; Amezrou et al., 2022). Though there are qualitative components to *Z. tritici* pathogenicity, it is regarded as being largely quantitative as phenotypes observed are mostly intermediate and do not correspond to a typically qualitative black or white situation (Hartmann et al., 2017; Stewart et al., 2018). Quantitative components of pathogenicity can be evaluated using different quantitative traits such as infection efficiency, latency period, pycnidia density, spore production, duration of the infectious period and lesion size (Pariaud et al., 2009; Lannou, 2012; Gohari et al., 2015; Stewart et al., 2018).

The only other gene-for-gene interaction that has been demonstrated is between *Stb7* or *Stb12* and *Avr3D1* (Meile et al., 2018). This interaction was shown to be linked to quantitative phenotypes despite the involvement of major resistance genes (Meile et al., 2018), a first for this pathosystem. Both *AvrStb6* and *Avr3D1* encode SSP (Zhong et al., 2017; Meile et al., 2018), and *AvrStb9* encodes a large, secreted protein with a protease-like domain (Amezrou et al., 2022). While the *Z. tritici* genome is composed of 13 core chromosomes, present in every known strain, and 8 accessory chromosomes, which are subject to presence/absence polymorphisms (Goodwin et al., 2011), to date, no QTL linked to pathogenicity have been identified on accessory chromosomes, though a small effect of these accessory chromosomes on pathogenicity has been detected (Habig et al., 2017).

Wheat cultivar ‘Renan’ displays quantitative resistance to *Z. tritici* strains I05 and I07 with three resistance QTL mapped on chromosomes 1D, 5D and 7B, the first two showing strain specificities between I05 and I07 (Langlands-Perry et al., 2022). We studied the progeny of a cross between the strains I05 and I07, aiming at deciphering the genetic architecture of pathogenicity in quantitative interactions and characterizing the underlying genes. We hypothesized that pathogenicity in this cross is also of quantitative and polygenic nature, with pathogenicity QTLs being involved in gene-for-gene interactions with the two strain-specific resistance QTL previously identified in Renan. We therefore aimed at demonstrating that gene-for-gene mechanisms do not exclusively underlie qualitative interactions, but are also involved in quantitative plant-pathogen interactions.

## 2 Materials and methods

### 2.1 Fungal material

The two *Z. tritici* strains “I05” (INRA09-FS0813, Mat1-1) and “I07” (INRA09-FS0732, Mat1-2), sampled in 2010 from STB lesions on wheat cv. Soissons in Grignon, France (48510 N, 1580 E), were crossed by co-inoculating adult plants with an equiproportional suspension of parental blastospores. After ascosporogenesis, 167 offspring individuals were collected from yeast-like colonies on Petri dishes placed upside down above wheat debris fragments to collect discharged ascospores as described in Suffert et al. (2016). The population of the 167 individuals resulting from the cross is hereafter referred to as “I05×I07”.

### 2.2 Inoculation procedure

148 chosen randomly among the I05×I07 progeny strains, and the two parental strains were phenotyped over three replications on 16-day-old seedlings of the wheat cultivar ‘Renan’ grown in a growth chamber as previously described in Langlands-Perry et al. (2022).

The strains were precultured in YPD (Yeast extract Peptone Dextrose), composed of 1% yeast extract, 2% peptone, 2% glucose and 95% distilled water, 10 days prior to inoculation, and each preculture was then grown on a PDA solid culture medium (potato dextrose agar), as described in Langlands-Perry et al. (2022). 150 mL of blastospores were prepared in advance from these cultures, each with a concentration of 10^6^ ± 1.10^5^ spores.mL^-1^, kept at -80°C between 1 to 3 months to be used for each of the three replicated inoculations. Before inoculation, a drop of Tween 20^®^ was added per 15 mL of inoculum to ensure adherence of the inoculum to the leaf.

The day before the inoculation, only three plants were kept per pot. On the first true leaf (about 3 cm from the base) of each plant, a surface of 7.5 cm in length was delineated with two black marks from a felt tip. Inoculation was carried out with cotton swabs, one per inoculum, in six passages (3 times back and forth), within the marks. After inoculation, the pot was covered with a plastic bag closed off at the top with a paper clip to not only create a water-saturated atmosphere, which encourages infection (Shaw, 1991; Boixel et al., 2022), but also to isolate the pots from one another to prevent cross contamination. The paper clips were removed after three days, a 72 h incubation period being the time it takes for the fungus to reach the mesophyll, which is necessary to the rest of the colonisation process (Kema et al., 1996). To optimise conditions for the survival of the inoculated leaf and to homogenise the quantity of light received by each leaf, new leaves were cut 2 to 3 cm above the first node 14 days post-inoculation (dpi).

### 2.3 Evaluation of phenotypic traits

#### 2.3.1 Visual evaluation of symptoms

The areas of the 7.5 cm-long inoculated leaf segment which were green, necrotic and sporulating (i.e. presented pycnidia) were assessed as percentages of the total inoculated area at 14, 20 and 26 dpi. The phenotypic traits S20 and S26 correspond to the percentage of the inoculated area presenting with pycnidia at 20 and 26 dpi, respectively. AUDPCs (Area Under the Disease Progress Curve) for the green, necrotic and sporulating areas (AUDPCG, AUDPCN and AUDPCS, respectively) were calculated as described by Langlands-Perry et al. (2022) using the three assessments realized over the course of the infection.

#### 2.3.2 Evaluation of sporulation by image analyses

Inoculated parts of the leaves were scanned and the images analysed with ImageJ, following the method of Stewart & McDonald (2014) and Stewart et al. (2016), modified by Langlands-Perry et al. (2022). The necrotic leaf surface and the total number of pycnidia were determined and used to calculate pycnidia density (PYC).

Sporulation for each three-leaf sample was quantified with the use of the particle size & shape analyser Occhio Flowcell FC200S+HR (www.occhio.be) according to the protocol followed by Langlands-Perry et al. (2022) and divided by the number of pycnidia of the three-leaf sample to calculate the number of pycnidiospores per pycnidium (NBS).

### 2.4 Statistical analysis of phenotypic data

The obtained data sets were analysed with the R software (R Core Team, 2019). For each trait an analysis of variance (ANOVA) was performed with the following model:


Yij=µ+Ii+rj+Irij+ϵij

Where Yij is the trait which is being studied, µ is the mean value for this trait,  Ii is the individual genotype, rj the replication, Irij the interaction and ϵij the residual. For the following analyses, Irij was included in the residual.

The following hypotheses were tested after the variance analyses.

$\epsilon \;∼\;N\left(o,\sigma^{2}\right)$

$cov\left(\epsilon ,\epsilon^{\prime }\right)=0\text{ Homoscedasticity (homogeneity of}var\left(\epsilon )\right)$

Broad-sense heritability is defined by the following formula:


$$H^{2}=\frac{\sigma g^{2}}{\sigma g^{2}+\sigma e^{2}}$$

Where $H^{2}$ is the heritability, $\sigma g^{2}$ the genotypic variance and $\sigma e^{2}$ the residual variance.

Two ANOVAs were carried out as 30 individuals were missing from the experiment for replicate 3. The first ANOVA included the data for all three replicates but with the 30 individuals missing from replicate 3 removed. The second ANOVA included the data for all individuals but only for the replicates 1 and 2.

The correlation between traits was studied using the Bravais-Pearson correlation.

### 2.5 RAD-sequencing of I05×I07 progeny isolates

The 167 progeny and two parental strains were grown over 7 days in a 250 mL Erlenmeyer flask containing 100 mL of YPD inoculated with 30 µL of inoculum. Growth chambers were fixed at 17°C, a hygrometry of 70%, constant agitation at 160 rpm and under neon lights (two Osram L 58W/840 Lumilux Cool White tubes). Then, spores were washed and transferred into 50 mL Falcon tubes to be lyophilized for 24 to 30 hours. The dry samples were ground in liquid nitrogen with a mortar and pestle. DNA was extracted following a phenol/chloroform-based protocol adapted from Fagundes et al. (2020). After drying, extracted DNA samples were suspended in 250 µL of Tris buffer at 10 mM. Sample purity was verified with a Nanodrop (Desjardins and Conklin, 2010) measurement while concentrations were measured with a Qubit 2.0 (Anon, 2011). All samples were subjected to an electrophoresis to verify that they were not degraded.

Samples were sequenced following the RADseq (Restriction site Associated DNA sequencing) strategy (Etter et al., 2011), on the Platform MGX (MGX-Montpellier GenomiX) on a HiSeq 2500 (Illumina) in paired-end 2*125nt mode. This type of sequencing enables one to target 1 to 10% of the genome *via* the use of a restriction enzyme and tagging of digested strands. Sequencing depth per sequenced locus is higher than classic sequencing, while the price is considerably lower. The restriction enzyme used was *Pst1*, following a previous study by Lendenmann et al. (2014), corresponding restriction sites were present throughout the IPO323 genome (**Figure S1**).

### 2.6 QTL mapping

A genetic map containing 18,316 SNP markers for 1,332 genetic bins covering the whole *Z. tritici* genome bar chromosomes 14 and 18 was built using the Multipoint ultra-dense software (MultiQTL Ltd, Haifa University, Israel) (details in Supporting information 2) and was used for QTL mapping.

A linkage analysis was carried out using the R/qtl software (Broman et al., 2003) version 1.42-8. This analysis included for each trait an initial Simple Interval Mapping (SIM), followed by a Composite Interval Mapping (CIM). Analyses were performed replication by replication. For SIM, 1000 genome-wide permutations were used to calculate the significant logarithm of odds (LOD) threshold. Were considered significant only QTL that showed P-values<0.05. The CIM was carried out with the QTL with the highest LOD used as a covariate. QTL intervals were evaluated with the LOD support interval with a drop in LOD of 1 and the “expandtomarkers” argument as true. QTL effects were calculated with the “effectplot” and “effectscan” functions. Possible epistatic interactions between QTL were looked into using the “addint” function.

### 2.7 Identification of a candidate pathogenicity gene

#### 2.7.1 QTL gene content

The gene content of the QTL identified on chromosome 6 was looked into using the annotation by Grandaubert et al. (2015). Certain reannotations were carried out using data from Haueisen et al. (2019) and RepeatMasker (Smit et al., 2013) for TE annotation. Functions of candidate genes were predicted using the translated protein sequences as input for the InterPro database (http://www.ebi.ac.uk/interpro/search/sequence/).

#### 2.7.2 Structural differences between I05 and I07 at the detected QTL

A *de novo* genome assembly of parental strains I05 and I07 was realized with previously available PE-100 sequences obtained on an Illumina HiSeq 2000 sequencing system with a 70x mean genome coverage (BioProject: PRJNA777581, accessions SRR16762604 and SRR16762605). Illumina paired-end reads were assembled using a combination of VELVET (Zerbino and Birney, 2008), SOAPDENOVO and SOAPGAPCLOSER (Luo et al., 2012), as previously described for the assembly of the *Botrytis cinerea* genome (Mercier et al., 2021).

The contigs from the I05 and I07 assemblies covering our regions of interest were identified by BLAST of the assembled genomes on the IPO323 reference genome (Goodwin et al., 2011). These enabled us to identify polymorphism between I05 and I07 for our candidate genes and TE presence/absence polymorphisms in both strains. The TE were annotated using ReapeatMasker (Smit et al., 2013) and a previously generated TE library for this organism (Grandaubert et al., 2015) according to the nomenclature defined by Wicker et al. (2007).

#### 2.7.3 Expression profiles for the top candidate gene

The relative expression of the candidate gene from *Qzt-I05-6* was assessed by analysing qPCR data following the 2^-ΔΔCt^ method (Livak and Schmittgen, 2001), the detail for which is in Supporting information 3 and all primers used are referenced in **Table S4**.

### 2.8 Molecular cloning

A detailed version is presented in Supporting information 4.

#### 2.8.1 Bacterial strains and DNA manipulation

For all PCR performed to obtain cloning fragments, the Taq polymerase Phusion^®^ (Thermo Fisher Scientific Inc., Waltham, MA, USA) was used under adapted PCR conditions using primers referenced in **Table S6**. All DNA assembly manipulations were conducted with the Gibson Assembly Cloning Kit (New England Biolabs, Ipswich, MA, USA). Plasmids carrying a hygromycin resistance gene flanked by two regions of approximately 1000 bp for homologous recombination were generated for knock-out mutants. Plasmids carrying a sulfonylurea resistance gene and a DNA fragment comprising at least 499 bp upstream of the start codon and at least 1 kb downstream of the stop codon of the candidate gene were generated for complementation and ectopic integration mutants. NEB 5-alpha Competent *Escherichia coli* (High Efficiency) (New England Biolabs, Ipswich, MA, USA) were transformed by heat shock with the generated plasmids and used for their amplification. Successfully transformed colonies were identified by PCR and mini-prepped plasmid constructs validated by Sanger sequencing (Eurofins, Luxembourg). *Agrobacterium tumefaciens* strain AGL1 was transformed by heat shock with each generated plasmid. Colonies were screened by PCR.

#### 2.8.2 *A. tumefaciens*-mediated transformation of *Z. tritici*

The *Z. tritici* strains I05 and I07 were transformed by ATMT (Bowler et al., 2010) following the standard protocol to generate knock-out mutants and ectopic integration mutants. This enabled us to obtain I05_ΔG07189 and I07_ΔG07189 mutants. I05_ΔG07189 was transformed following the same protocol to generate complementation mutants I05_ΔG07189 + G07189_I05_ and I05_ΔG07189 + G07189_I07_ and ectopic integration mutant I05 + G07189_I07_.

Mutant strains were selected by hygromycin or sulfonylurea screening. Obtained strains were verified by PCR on genomic DNA extracted with the DNeasy^®^ Plant Mini Kit (Qiagen, Hilden, Germany) according to the supplier’s protocol.

#### 2.8.3 Phenotypic characterization of mutant strains

All generated mutant strains were inoculated on ‘Renan’ and ‘Chinese Spring’ (susceptible control) according to the same protocol as the previously described assays. Only visual evaluations were performed. Three clones of each mutant strain type and I05 and I07 were tested, three times each per replication. Four replications were carried out. The results obtained were analysed with a Kruskal-Wallis test and a Wilcoxon pairwise comparison.

### 2.9 Diversity and selection analysis of the candidate effector from Qzt-I05-6

In addition to the I05 and I07 *de novo* assemblies, the assemblies of 103 *Z. tritici* strains collected in France in 2009-2010 (BioProject: PRJNA777581) and 126 strains collected in France in 2018-2019 (BioProject: PRJNA881220) were also available to us, enabling us to look into the sequence diversity of the pathogenicity gene. We extracted gene sequences from genome assemblies of the 229 *Z. tritici* strains using the ncbi-blast+ software (Camacho et al., 2009). Prior to performing the population genetic analysis, we first verified population structure in our dataset, to ascertain that there is no inflation due to population structure. We constructed a protein sequence phylogenetic tree using the RaxML algorithm with the GAMMA JTT model and 100 bootstrap replicates (Stamatakis, 2014). Phylogenetic trees were visualized using iTOL (Letunic and Bork, 2016); https://itol.embl.de/ . We used the R package Popgenome (Pfeifer et al., 2014) to calculate sliding-window analyses of nucleotide diversity (π) of the effector gene, including ~500 bp upstream and downstream of the coding sequence. We used a window length of 20 bp and a step size of 5 bp. To verify whether the effector gene exhibits signatures of positive diversifying selection, we calculated the rates of ω, the ratio of nonsynonymous to synonymous mutational rates using the codon-based selection analysis codeML (Yang et al., 2004). The ratio indicates negative purifying selection (0< ω< 1), neutral evolution (ω = 1), or positive diversifying selection (ω > 1). We compared different evolutionary models and used the statistical likelihood ratio test (LRT) to determine the model that best fitted our data. The Bayes empirical Bayes method (BEB) was then used to evaluate the posterior probability of sites considered to have been positively selected.

## 3 Results

### 3.1 Phenotypic data analysis

A representation of the distribution of the different traits for all three replicates shows that they do not follow a normal distribution (**Figure S2**), as confirmed by a Shapiro-Wilk normality test. For all traits, transgressive individuals are observed.

**Figure 1:**
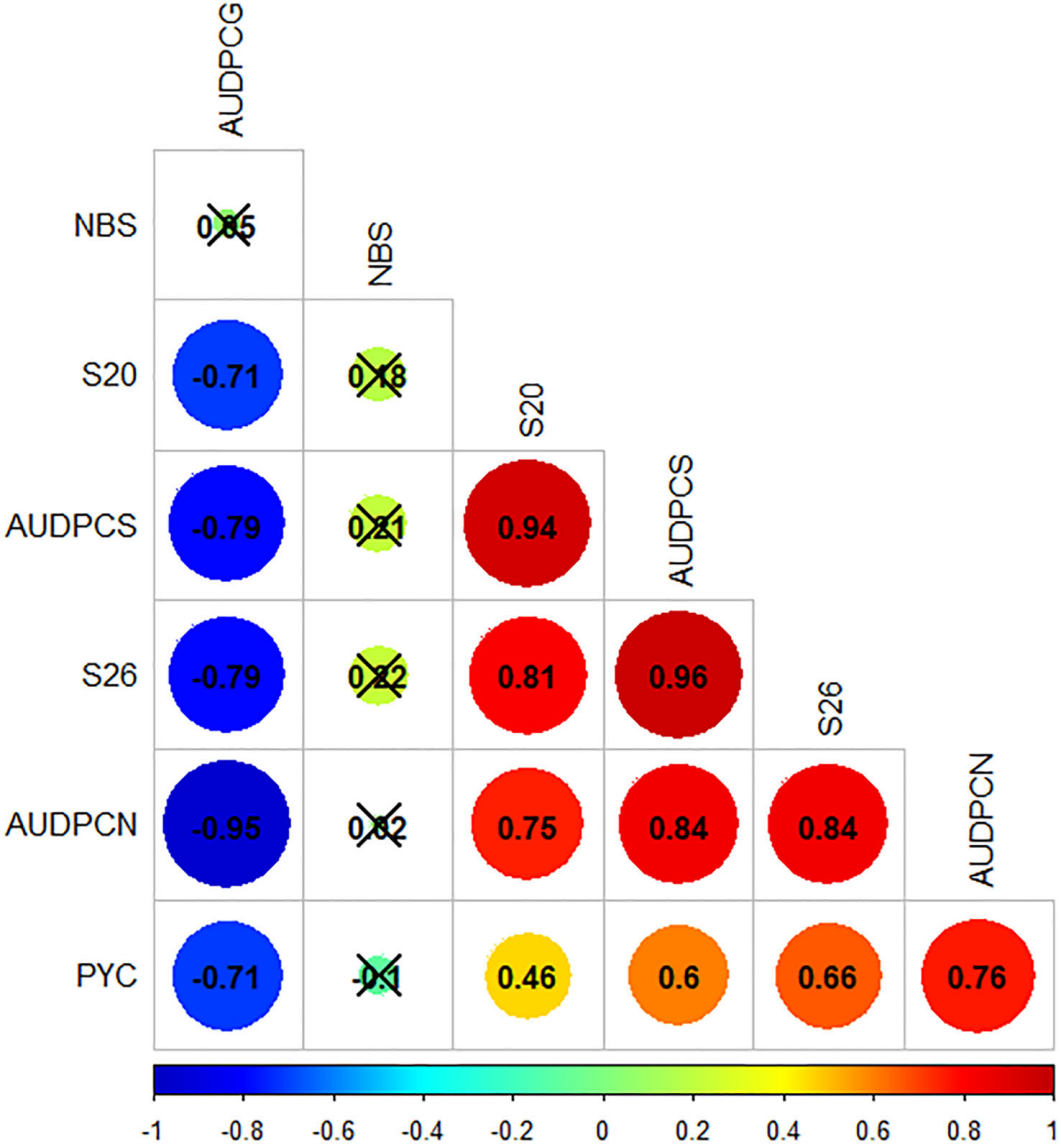
Bravais-Pearson correlogram for the phenotypic data obtained from the inoculation of the *Zymoseptoria tritici* I05xI07 progeny strains on the wheat cultivar ‘Renan’. Crossed out correlation values are not statistically significant (p-value<0.05). PYC is the pycnidia density. NBS is the number of spores per pycnidiospore. AUDPCs are the Area Under the Disease Progress Curve for the green, necrotic and sporulating areas (AUDPCG, AUDPCN and AUDPCS, respectively). S20 and S26 are the sporulating leaf area at 20 and 26 days post-inoculation, respectively.

Bravais-Pearson correlation results show that all traits but NBS were correlated (**Figure 1**). The most strongly correlated traits were S26 and AUDPCS with a correlation coefficient of 0.96, both traits linked to the sporulating area. AUDPCN and AUDPCG were also strongly negatively correlated with a correlation coefficient of -0.95. PYC was less strongly correlated overall than the other traits, it was however significantly correlated, with absolute correlation coefficient values ranging from 0.46 to 0.76 for PYC (NBS values excluded).

Both ANOVAs (**Table S3**) showed that the genotype had a significant effect on phenotypes, though the significance was milder for PYC and NBS. They also showed that, overall, the replication had high statistical significance. This led to all subsequent analyses being carried out replication by replication. The traits that best performed in the statistical analyses were AUDPCG, AUDPCN and PYC with the ANOVA assumptions respected. For the other traits, the assumptions were not so well respected and heritability may not be optimally estimated. Heritability ranged from 0.45 to 0.59 for S26, AUDPCG, AUDPCN and AUDPCS, values that are reasonably high. The values were lower for S20, PYC and NBS, ranging from 0.16 to 0.36.

### 3.2 Linkage analyses of I05×I07 progeny reveal three pathogenicity QTL

**Figure 2:**
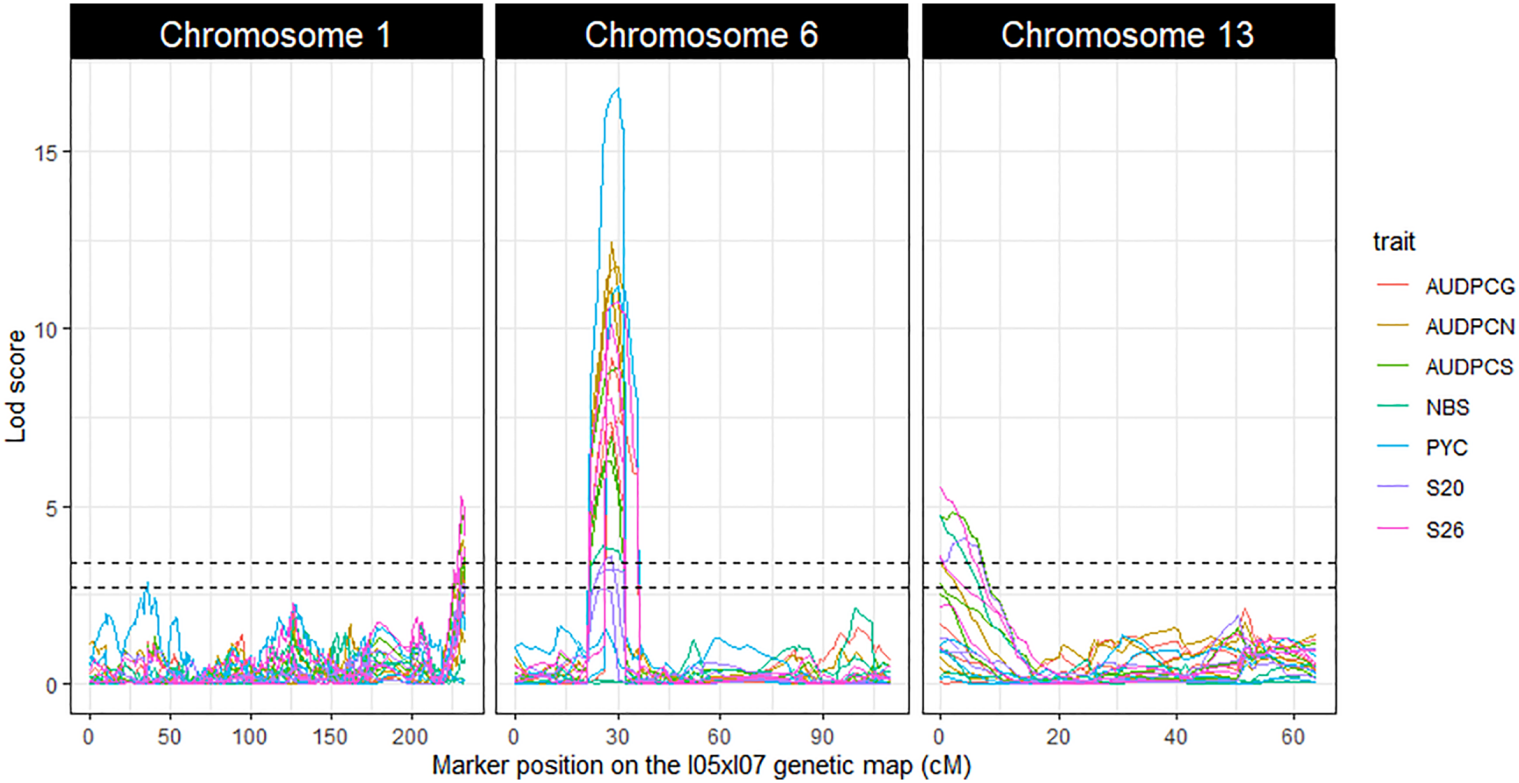
LOD score profiles for the linkage analyses performed on the phenotypic and genetic data generated from the *Zymoseptoria tritici* I05xI07 population. The X-axis represents the position of the markers on the I05xI07 genetic map in cM. The Y-axis represents the LOD score associated with the markers. Dotted lines represent the minimal and maximal LOD threshold values obtained in the linkage analyses after 1000 permutation tests. Each column corresponds to a chromosome, chromosomes 1, 6 and 13 respectively. The colours in the graphs correspond to the studied traits. AUDPCs are the Area Under the Disease Progress Curve for the green, necrotic and sporulating areas (AUDPCG, AUDPCN and AUDPCS, respectively). S20 and S26 are the sporulating areas (%) at 20 and 26 days post-inoculation, respectively.

The linkage analyses enabled us to detect three repeatable QTL: *Qzt-I05-1*, *Qzt-I05-6* and *Qzt-I07-13*, on core chromosomes 1, 6 and 13, respectively (**Table 1**, **Figure 2**, **Table S7**). *Qzt-I05-1* and *Qzt-I05-6* were detected for all three replicates, while *Qzt-I07-13* was detected only for replicates 2 and 3 (**Table 1**). *Qzt-I05-1* is in a sub-telomeric region of chromosome 1. It covers a 4.96 cM-long region on the genetic map spanning 138 kb based on the physical position of the flanking markers. The mean phenotypic variation explained by this QTL is 6.37% and the parental strain carrying the pathogenic allele for this QTL is I05.

**Table 1:** QTL for pathogenicity detected through linkage analysis using the phenotypic data and the genetic map generated from the *Zymoseptoria tritici* I05xI07 population inoculated on wheat cultivar ‘Renan’.

L	Number of detections	Maximal phenotypic variance (%)	Mean phenotypic variance (%)	Parent carrying the pathogenic allele	Traits^1^
*Qzt-I05-1*	8	7.43	6.37	I05	S26, AUDPCG, AUDPCN, AUDPCS
*Qzt-I05-6*	17	40.15	24.91	I05	S20, S26, AUDPCG, AUDPCN, AUDPCS, PYC, NBS
*Qzt-I07-13*	6	9.40	6.93	I07	S20, S26, AUDPCN, AUDPCS, NBS

^1^AUDPCs are the Area Under the Disease Progress Curve for the green, necrotic and sporulating areas (AUDPCG, AUDPCN and AUDPCS respectively). S20 and S26 are the sporulating areas (%) at 20 and 26 days post-inoculation respectively. PYC is the pycnidia density. NBS is the number of pycnidiospores per pycnidium.

*Qzt-I05-6* covers a 13.48 cM-long region corresponding to a physical interval of 169 kb on chromosome 6. The mean phenotypic variation explained by this QTL is 24.91%, the highest among the three repeatable QTL detected, and the parental strain carrying the pathogenic allele for this QTL is I05. This QTL presents itself as being the most robust of the three.

Finally, *Qzt-I07-13* is found at a sub-telomeric region of chromosome 13. It covers an 8.64 cM-long region corresponding to a physical interval of 186 kb. The mean phenotypic variation explained by this QTL is 6.93% and, contrary to the other two QTL, the parental strain carrying the pathogenic allele is I07.

**Figure 3:**
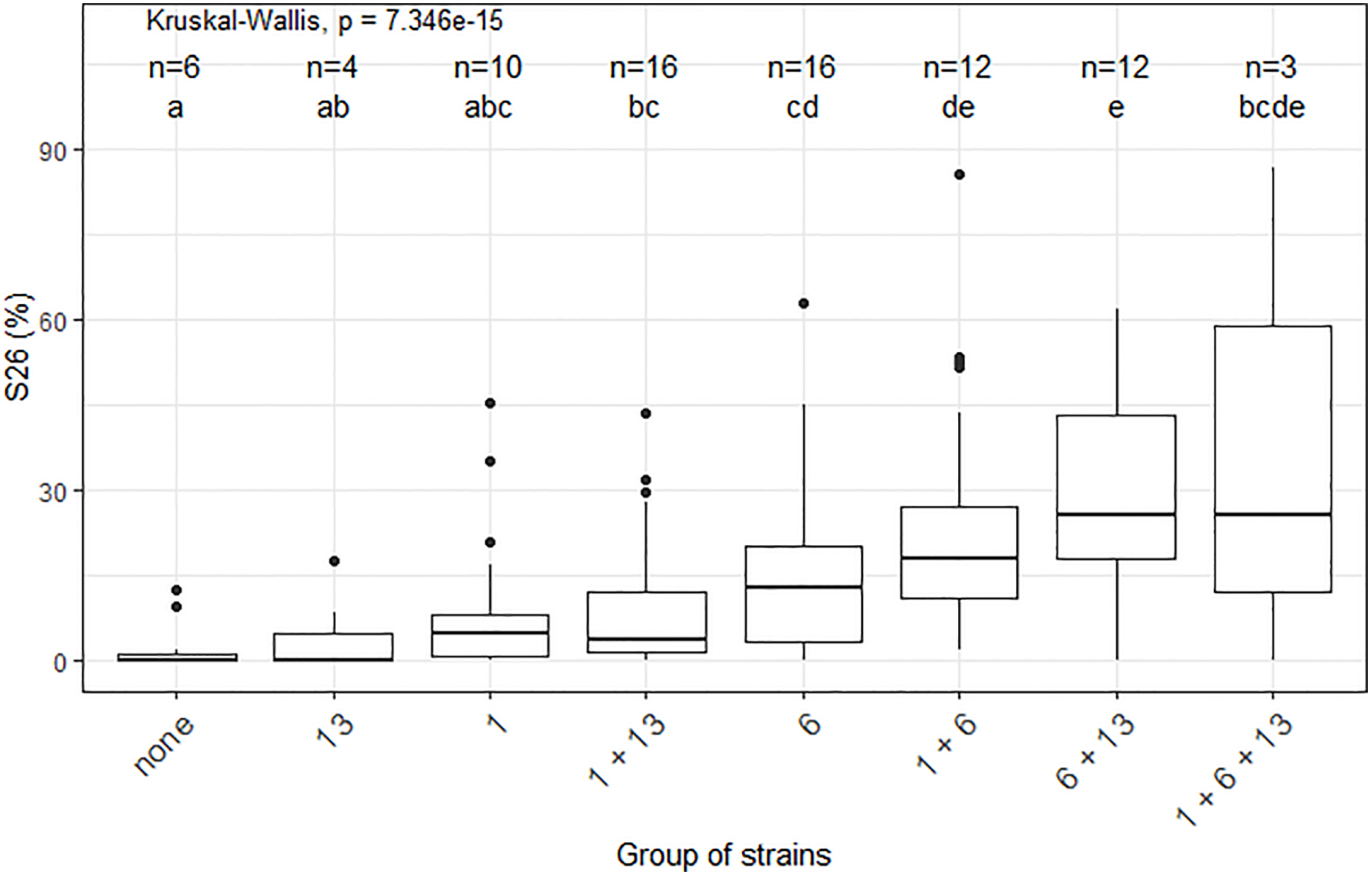
Impact of the different combinations of pathogenicity QTL on the performance of strains from the *Zymoseptoria tritici* I05×I07 population on the wheat cultivar ‘Renan’, strains with recombination within the QTL intervals were excluded. The X-axis represents the different groups of strains concerning their pathogenic allele combination per QTL. For legibility, the QTL are referred to here as 1, 6 and 13. The n values correspond to the numbers of strains per group. The Y-axis represents the phenotypic values obtained. S26 is the sporulating area (%) at 26 days post-inoculation. The Kruskal-Wallis values indicate that the phenotypic value for at least one group of strains is significantly different to the others. Letters a, b, c, d and e indicate a significant difference for a Wilcoxon pairwise comparison at alpha=0.05.

Significant epistatic interactions were detected between *Qzt-I05-1* and *Qzt-I05-6* and between *Qzt-I05-6* and *Qzt-I07-13* (**Table 2**). *Qzt-I05-6* and *Qzt-I07-13* had the highest and most significant epistatic interaction explaining on average 7.12% of phenotypic variation. This epistatic interaction explains why strains carrying the pathogenic allele for these two QTL led to the highest S26 values among the strains combining the pathogenic allele for two QTL (**Figure 3**).

**Table 2:** Epistatic interactions detected between the QTL identified with the *Zymoseptoria tritici* I05xI07 population inoculated on wheat cultivar ‘Renan’.

Replication	Traits^1^	Chromosomes on which QTL were detected	Range of phenotypic variance due to epistatic effect (%)^2^
1	AUDPCS, S26	1, 6	5.19***-5.8***
2	AUDPCS, S26	1, 6	2.72*-2.74*
3	AUDPCN, AUDPCS	1, 6	2.73*-6.59***
2	S26	6, 13	3.62**
3	AUDPCN, AUDPCS, S20, S26	6, 13	4.07**-10.8***

^1^AUDPCs are the Area Under the Disease Progress Curve for the green, necrotic and sporulating areas (AUDPCG, AUDPCN and AUDPCS respectively). S20 and S26 are the sporulating areas (%) at 20 and 26 days post-inoculation respectively. PYC is the pycnidia density. NBS is the number of pycnidiospores per pycnidium.

^2^Significance codes: 0 ‘***’, 0.001 ‘**’, 0.01 ‘*’. Detected interactions with very low significance are not shown.

For further investigation, we chose to focus our efforts on *Qzt-I05-6* which was the most robust QTL, with the strongest effect on phenotypic variation.

### 3.3 Qzt-I05-6 harbours a previously unannotated effector-like gene in a dynamic region

The interval defined by *Qzt-I05-6* holds 36 genes annotated in Grandaubert et al. (2015). There is a 60 Kb-long TE-rich region in the middle of the QTL, with markers that came out as peak markers during the linkage analysis on either side. None of these 36 genes has any predicted functions; we therefore predicted functions with the InterPro database (http://www.ebi.ac.uk/interpro/search/sequence/) (**Table S8**). We searched for genes that have characteristics of known effectors, as these are often involved in fungal pathogenicity. Out of the 36 annotated genes, two had a signal peptide. The first, named *Zt09_6_00095* in the Grandaubert et al. (2015) annotation, comes out as being in the PTHR35523 family in the panther classification system (http://www.pantherdb.org/), which regroups cell wall proteins. As a component of the cell structure, this gene does not seem to be a good candidate. The second gene, *Zt09_6_00123*, is predicted to encode a FAD binding domain, linking it to an oxidoreductase process. This gene is a little large for an effector with a length of 1272 bp and a corresponding protein of 423 amino acids (aa), the general cut-off being set at 300 aa (Sperschneider et al., 2018). It has a predicted function and only one cysteine in its protein sequence. It does not seem to be a good candidate either.

RNAseq data from Haueisen et al. (2019) enabled us to notice some reads that mapped to a position that was not annotated, right next to the central TE region. As pathogenicity genes can be found in regions such as this (Plissonneau et al., 2018), we looked further into the corresponding position. The RNAseq data produced by Haueisen et al. (2019) was used to make a read coverage file which was used in the integrative genomics viewer (IGV) software (Robinson et al., 2011) as a means of annotating a previously unidentified gene. On chromosome 6 of IPO323, this gene is positioned at 470,027-470,324 bp. It has two exons with coding sequence (CDS); the first has a predicted signal peptide (https://services.healthtech.dtu.dk/service.php?SignalP) (Teufel et al., 2022), and there is no predicted function or family for the protein (http://www.ebi.ac.uk/interpro/search/sequence/). It has an intron which is 61 bp long and has canonical splice site combination GT-AG (Kupfer et al., 2004; Frey and Pucker, 2020). The CDS is 237 bp long and encodes a 78-aa protein with 11 cysteines in its sequence (14% of the protein sequence). We identified four mutations between parental strains I05 and I07. In a recent, yet unreleased, annotation of the *Z. tritici* IPO323 genome sequence, specifically improved to detect genes encoding SSP, this gene was identified and named *G_07189* (unpublished data). A BLAST search against the NCBI database (https://blast.ncbi.nlm.nih.gov/Blast.cgi) identified in other *Z. tritici* strains a second, longer protein, with a potential alternative methionine 32 aa before the predicted one. This longer, alternative protein has a cleavage site between the 47^th^ and 48^th^ aa, that is unlikely for a signal peptide. Searches for a GPI anchor did not return positive results. Therefore, the shortest isoform of 78 aa was retained for *G_07189*. The BLAST search did not yield any significant results in other species, indicating that this gene is species specific.

**Figure 4:**
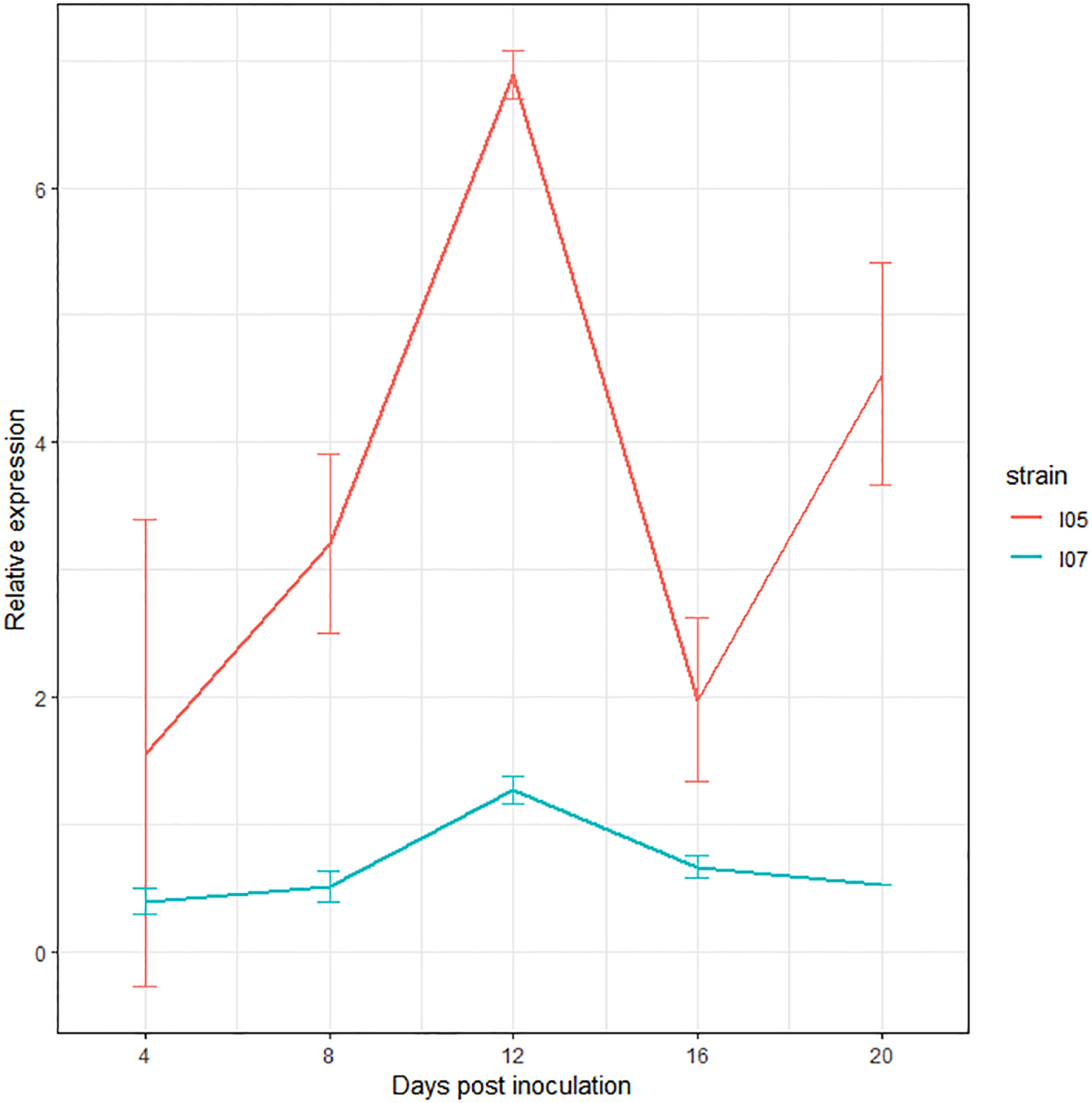
Expression profile of *G_07189* in *Zymoseptoria tritici* strains I05 (red) and I07 (blue) during infection on the *T. aestivum* cultivar ‘Chinese Spring’. The values shown are the relative expression levels for each gene with respect to the geometric mean obtained for the three housekeeping genes used (*EF1α*, *UBC* and *β-tubuline*), averaged over at least two biological replicates, except for I07 at 20 dpi, where all samples but one were degraded after RNA extraction and DNase treatment. Error bars represent the 95% confidence intervals of the averages.

The study of the expression of this gene by RT-qPCR over the course of the infection revealed upregulation at 12 dpi in both I05 and I07 strains (**Figure 4**), further consolidating its status as a very good effector candidate.

**Figure 5:**
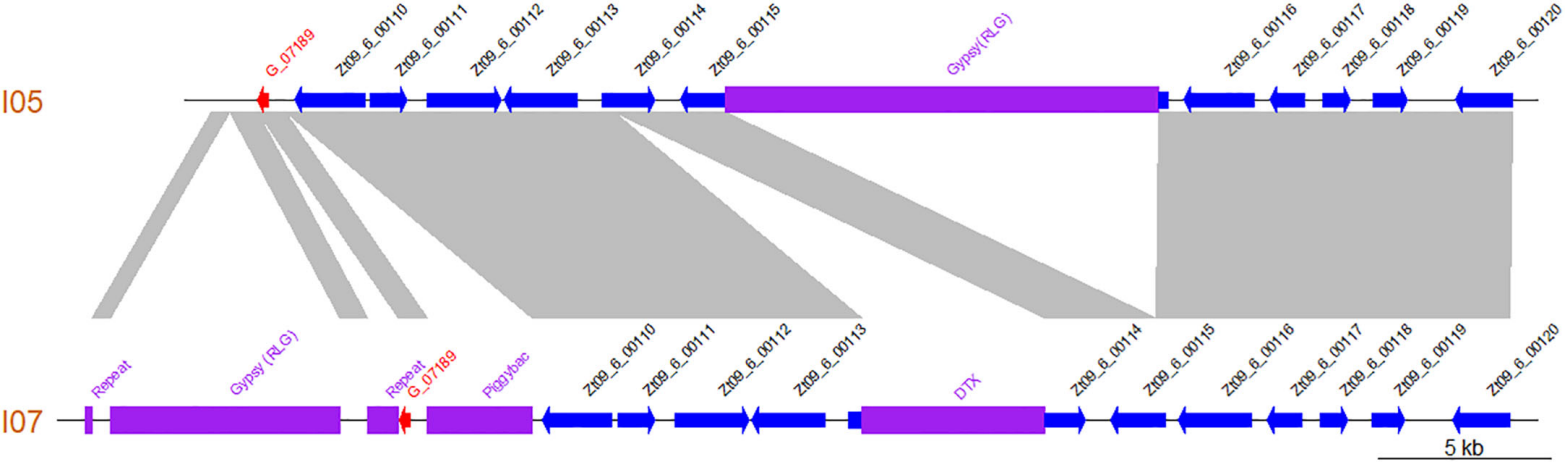
The region harbouring gene *G_07189* is highly polymorphic between *Zymoseptoria tritici* strains I05 and I07 as illustrated by this synteny plot comparing the contigs of the I05 and I07 assemblies which carry *G_07189*. Previously annotated genes are represented as blue arrows according to their orientation. The newly annotated gene *G_07189* is represented by a red arrow according to its orientation. Transposable elements are represented by purple blocks. Collinear sequences between contigs are shown in grey.

Additionally, comparison of the I05 and I07 assembled contigs revealed that the newly annotated gene lies in a highly dynamic region, with several presence/absence polymorphisms of TE between both parental strains (**Figure 5**).

### 3.4 Genetic manipulation and pathology tests validate the involvement of G_07189 in pathogenicity

All knock-out, complementation and ectopic integration mutant strains were successfully generated following an ATMT protocol (Bowler et al., 2010). Three randomly selected mutants per construction were able to successfully infect ‘Chinese Spring’ and induce sporulation covering on average 99% of the inoculated leaf area (**Figure S4**).

**Figure 6:**
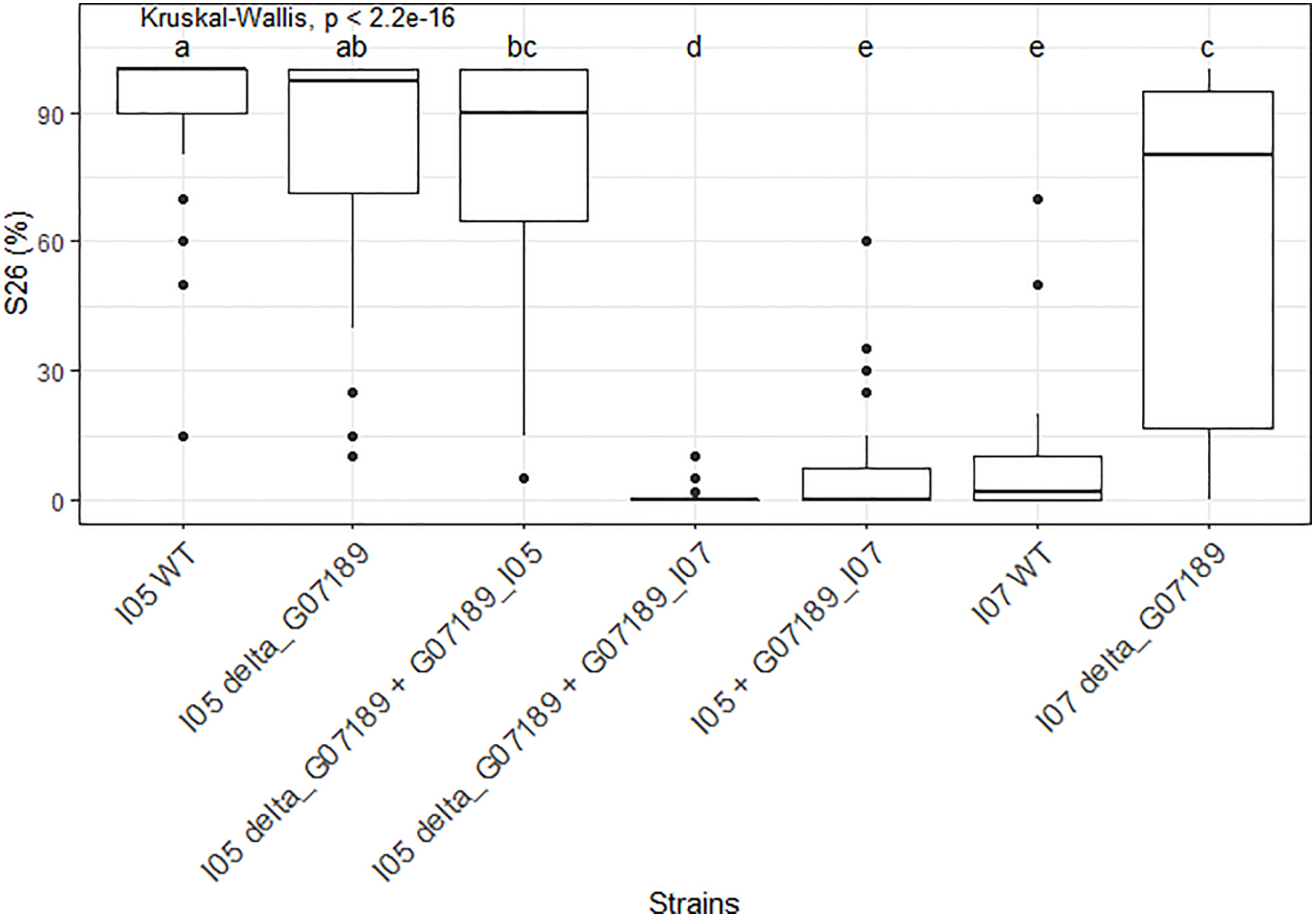
S26, the sporulating area (%) at 26 days post-inoculation, values observed for the different mutant *Zymoseptoria tritici* strains obtained for gene *G_07189* inoculated on wheat cultivar ‘Renan’. The Kruskal-Wallis value indicates that the phenotypic value for at least one type of strain is significantly different to the others. Letters a, b, c, d and e indicate a significant difference for a Wilcoxon pairwise comparison at alpha=0.05. The X-axis represents the different mutant strains tested. The Y-axis represents the phenotypic values obtained. The phenotypic data presented comprises all replicates for all individuals; the detail per individual is available in **Supplementary Figure S4**.

In the I05 genetic background, deleting gene *G_07189* has no effect on the phenotype, while replacing the I05 allele (G07189_I05_) by the I07 allele (G07189_I07_) induces a suppression of sporulation (**Figure 6**). Indeed, the I05 wild type protein induces a highly susceptible reaction on wheat cultivar ‘Renan’ (>90% sporulating leaf area) while ‘Renan’ becomes completely resistant when the I07 allele is introduced (**Figure 6**). In the I05 genetic background, the I07 allele of *G_07189* behaves as a classic avirulence gene inducing complete resistance in the host.

In the I07 genetic background, deleting the gene *G_07189* also has a strong and significant effect on sporulation as I07 wild type induces 7% sporulating leaf area on average while the knock-out mutants I07_ΔG07189 induce 61% sporulating leaf area on average (**Figure 6**). In the I07 genetic background, the effect of the I07 allele of *G_07189* remains quantitative.

### 3.5 G_07189 is highly conserved in French populations of *Z. tritici* but is under diversifying selection

**Figure 7:**
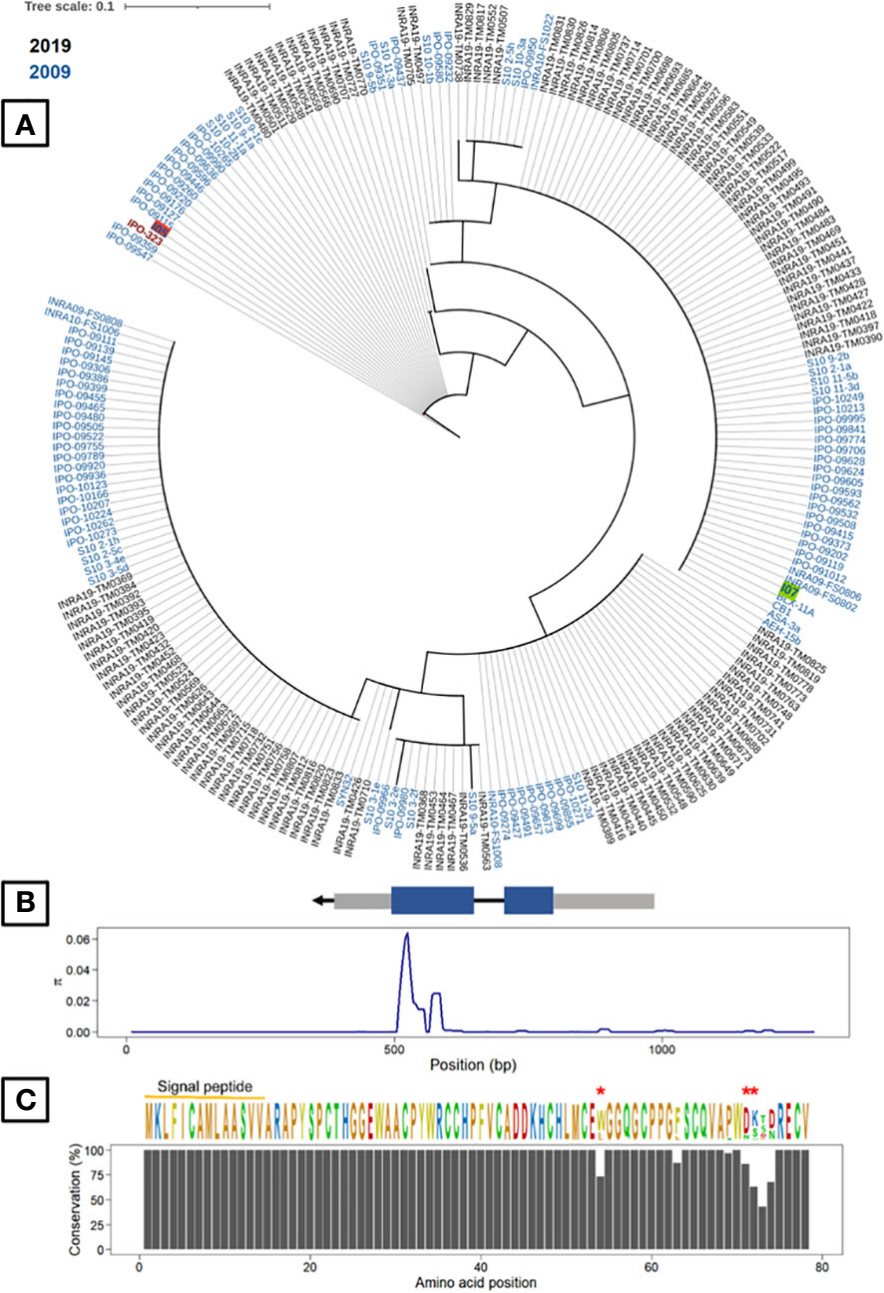
Gene *G_07189* in *Zymoseptoria tritici* is highly conserved and exhibits signatures of diversifying selection. **(A)** Phylogenetic tree of predicted Avr generated from protein sequences of two *Z. tritici* French collections and the reference isolate IPO323. The least pathogenic (I07) and most pathogenic parents (I05) are highlighted in green and red, respectively. **(B)** Top: Avr gene structure including untranslated regions or UTRs (grey boxes) and the DNA coding sequence (CDS) of the mature protein (blue boxes). Bottom: Sliding-window analyses of nucleotide diversity (π). **(C)** Top: The consensus sequence and sequence logos of Avr in the fungal population. Residues under significant diversifying selection (p<0.01) are highlighted with red asterisks. Bottom: Conservation of Avr protein residues.

As the cloning experiments validated the effect of *G_07189*, we analysed its genetic diversity in the 229 French *Z. tritici* strains and searched for diversifying selection signatures. This analysis showed that *G_07189* is highly conserved among these strains, regardless of their being collected in 2009-2010 or in 2018-2019, with non-synonymous polymorphism for only 7 amino-acid residues out of 78 (**Figure 7**). With no collection period-related population structure, the strains carry alleles encoding fifteen isoforms of the *G_07189* protein with four isoforms representing 86% of all strains including the I05 and I07 isoforms (**Figure 7**). All of the sequence diversity observed for the gene is coded by the second exon, with π_CDS2 =_ 0.02254 while π_CDS1_ and π_intron_ are around 0.0001 (**Table S9**). The ratio between non-synonymous and synonymous mutations (ω=dN/dS) in the panel of strains was calculated at 2.118 for a one-ratio model M0 (**Table S10**), a high value indicating diversifying selection, further validated by the selection M2 model having the highest InL value (-487.686, p<0.001) among the three models tested (**Table S10**). According to the codon-based maximum likelihood approach, three residues are under significant diversifying selection (p<0.01) at positions 54, 71 and 72 in the protein sequence (**Figure 7**). We therefore have a highly conserved gene that is under diversifying selection.

## 4 Discussion

### 4.1 The interaction between ‘Renan’ and I05×I07 progeny is polygenic and quantitative

In this study we identified regions in the *Z. tritici* genome, which carry genes that contribute to quantitative pathogenicity towards *T. aestivum*. The cultivar used was ‘Renan’, known to carry at least three resistance QTL with different levels of resistance towards I05 and I07 on chromosomes 1D, 5D and 7B (Langlands-Perry et al., 2022). Additionally, the resistance QTL on ‘Renan’ show strain specificity between I05 and I07 and none of these QTL alone is able to confer total resistance to either I05 or I07, indicating that resistance is polygenic and quantitative in the ‘Renan’/I05-I07 interaction (Langlands-Perry et al., 2022). Pathogenicity towards ‘Renan’ is polygenic, with three QTL identified in this study, each contributing partially to the observed phenotypic variation. For two of these QTL, the parent carrying the pathogenic allele is I05, the parental strain known to be the most pathogenic on ‘Renan’. For the third, the parent carrying the pathogenic allele is I07, the least pathogenic parental strain on ‘Renan’. We showed that these QTL have varying effects on the phenotype, with *Qzt-I05-6* having the strongest effect, and are subject to epistatic interactions, notably in the case of the *Qzt-I05-6* and *Qzt-I07-13* interaction. None of the detected QTL colocalizes with an already known avirulence gene in *Z. tritici*. Indeed, *AvrStb6* is found on chromosome 5 (Zhong et al., 2017), *AvrStb9* is on chromosome 1 but not in the region defined by *Qzt-I05-1* (Amezrou et al., 2022) and *Avr3D1* is on chromosome 7 (Meile et al., 2018). Looking at progeny strains that did not have any recombination within the QTL intervals, we are able to separate the different strains according to the QTL allele combination that they carry to observe the impact of these allele combinations on the performance of the strains on ‘Renan’ (**Figure 3**). We looked into the data for maximal sporulation S26 as this trait led to the detection of all three QTL. S26 was always higher for strains with the pathogenic allele for several QTL compared with strains carrying the pathogenic allele for only one QTL, though the difference was not always statistically significant, indicating incomplete additivity of the QTL effects. None of the non-pathogenic alleles of the QTL induced a completely resistant phenotype such as that conferred by a major gene like *Stb6* in the presence of *AvrStb6* (Zhong et al., 2017). The only strains that were almost completely non-pathogenic were those that carried the non-pathogenic allele for all three QTL, demonstrating the quantitative and polygenic nature of pathogenicity and mirroring the quantitative and polygenic nature of resistance in ‘Renan’ (Langlands-Perry et al., 2022).

### 4.2 Fungal effectors involved in quantitative interactions

An effector-like gene (i.e. coding for SSP) was identified as a candidate to explain *Qzt-I05-6*. This gene had not been previously annotated but was supported by RNA sequences obtained from infected leaves (Grandaubert et al., 2015; Haueisen et al., 2019). Moreover, in a recent reannotation of the *Z. tritici* genome, which aimed at optimizing the detection of SSP (unpublished data), this gene was annotated and named *G_07189*. Additionally, qPCR performed to evaluate relative expression of the gene over the course of infection showed differential expression patterns, with an expression peak at 12 dpi further validating its status as a good effector candidate.

We were able to demonstrate the role of *G_07189* in the pathogenicity of I05 and I07 on the cultivar ‘Renan’. The knock-out of this gene from I05 (most pathogenic parental strain on ‘Renan’) which generated I05_ΔG07189 strains, did not have a significant effect on the phenotype. The inclusion of the I07 (least pathogenic parental strain on ‘Renan’) allele for this gene, generating strains I05_ΔG07189+G07189_I07_ and I05+G07189_I07_, led to significantly lower S26 regardless of whether or not the I05 allele was present in the genetic background. The same phenomena were observed in the characterization of *Avr3D1* (Meile et al., 2018), where the *Avr3D1* knock-out in the background of the most pathogenic parental strain of the pair in that study (3D7) was unaltered compared to the wild-type. The knock-out however led to increased disease symptoms in the background of the strain with lower pathogenicity used in that study (3D1) compared to the wild-type.

This, corroborated with our results, suggests that *G_07189* encodes an avirulence factor. Additionally we showed that the region in which *G_07189* lies is TE rich and presents with TE presence/absence polymorphisms (**Figure 5**), similar to what has been previously observed with *AvrStb6* and *Avr3D1* (Zhong et al., 2017; Meile et al., 2018). Moreover, while I07, which carries the avirulent allele for *G_07189*, is able to produce symptoms on ‘Renan’ (i.e. S26 averaging at 10%), I05_ΔG07189+G07189_I07_ strains, which all carry this same allele, do not produce sporulation on ‘Renan’ (i.e. S26 averaging at 0.8%). *G_07189* therefore leads to a quantitative phenotype in the I07 background, but to a qualitative, avirulent, phenotype in the I05 background. Rather than acting like a classic avirulence gene in an *R*/*Avr* interaction, *G_07189* has a spectrum of effects depending on the genetic background of the strain. This is illustrated in **Figure 3**, where the strains in the “none”, “13”, “1” and “1+13” categories, which all carry the avirulent allele for *G_07189*, have different effects, if not always significantly, on the phenotype. These different effects form a gradient depending on the QTL combinations. Such a gradient in phenotypes, or variation of magnitude of the effect of quantitative pathogenicity on the phenotype, was recently demonstrated to also occur depending on the allele of the *Avr* gene in an isogenic background (Meile et al., 2023).

We are able to present conclusive evidence that an *Avr* gene can have a quantitative or qualitative effect on the phenotypes depending on the fungal genetic background. Pathogenicity is therefore not a strictly quantitative or qualitative variable, but rather fits somewhere on the continuum that they form. Recently, another example of an avirulence factor being involved in a quantitative interaction was proposed within the *Leptosphaeria maculans*-rapeseed pathosystem (Jiquel et al., 2021). A gene-for-gene interaction was demonstrated between LmSTEE98, a ‘late effector’ encoded by *AvrLmSTEE98*, and RlmSTEE98, encoded by the corresponding resistance gene. As a ‘late effector’, *AvrLmSTEE98* is expressed during stem colonization which is considered to be polygenic and quantitative.

On the plant side, it has been previously suggested that quantitative resistance genes in plants could be weaker forms of major resistance *R* genes (Poland et al., 2009) and that most wheat resistance loci are matched by a specific effector (Plissonneau et al., 2018). We therefore propose that *G_07189* could interact with the wheat resistance QTL identified in ‘Renan’ on chromosome 5D, designated as *Stb20q*, as this was the only QTL identified in ‘Renan’ which had specific resistance to I07 (Langlands-Perry et al., 2022). I07 carries the avirulent allele for *G_01789*, meaning that this gene could correspond to *AvrStb20q*.

### 4.3 Quantitative resistance durability despite gene-for-gene interactions

In a classic *R*/*Avr* gene-for-gene relationship, disease resistance is not considered durable due to the high probability of adaptation by the fungus with mutations in the *Avr* gene (Niks et al., 2011; Hartmann et al., 2017; Meile et al., 2018; Cowger and Brown, 2019). Here, we observed no loss of *G_07189* in the fungal populations or indeed any mutations in the effector-like features of the corresponding protein, i.e., the signal peptide and cysteine residues, inferring a potential fitness cost of such a loss. We found that the validated effector *G_07189* is highly conserved among French *Z. tritici* strains and shows a signature of positive diversifying selection. This is also what was observed for *Avr3D1*, the only other cloned effector in this pathosystem known to be involved in quantitative interactions (Meile et al., 2018). In the latter case however, a high level of diversity was observed in fungal populations. *G_07189* has the capacity to evolve to adapt to wheat resistance, as demonstrated by the diversifying selection signatures in three residues. The low diversity and fact that the virulent, or most pathogenic, isoform (I05), is not the most abundant in recent strains may therefore seem paradoxical. Two potential explanations for this paradox are, first, that the corresponding resistance gene, which we hypothesize to be *Stb20q*, has not been largely deployed in the French cultivar landscape, meaning that no selection due to this resistance has been imposed on fungal populations. The cultivar on which *Stb20q* was identified is ‘Renan’ (Langlands-Perry et al., 2022), mostly used in organic farming, and therefore not one of the majority cultivars in the landscape. The other explanation is that the selective pressure imposed on *G_07189*, despite being present, is low enough that adaptation is slowed down compared to what is observed with major *R*/*Avr* interactions. Indeed, the least pathogenic allele (I07) does not stop the fungus from completing its life cycle, as illustrated by the presence of pycnidia on the infected leaf surface. This second hypothesis fits in with the narrative that quantitative resistance is more durable than its qualitative counterpart.

An example of a highly conserved effector under diversifying selection is APikL2 in *M. Oryzae* (Bentham et al., 2021). Research has shown that a single amino-acid polymorphism is sufficient to evade host recognition, however, the evolutionary driver of this polymorphism was attributed to the expansion of the host target spectrum rather than immune receptor evasion (Bentham et al., 2021). Moreover, similar to what we observe with *G_07189*, diversifying residues are located at a specific part of the protein (Bentham et al., 2021). This example opens a new avenue of possibilities as to the molecular interactions in which *G_07189* could be involved.

## 5 Conclusion

This study confirmed that pathogenicity in *Z. tritici* is complex and largely quantitative. We showed that several genes underlying QTL interact and contribute to the *T. aestivum* infection with varied impact on the phenotype. We demonstrated that genes underlying pathogenicity QTL can be effectors or *Avr* genes. These *Avr* genes can produce quantitative or qualitative phenotypes depending on the genetic background of the strains that carry them, advocating for a continuum between qualitative and quantitative notions of pathogenicity. We hypothesize the involvement of these effectors in minor gene-for-minor gene interactions, although this remains to be experimentally validated, in particular in the case of the putative *Stb20q*/*AvrStb20q* interaction. Furthermore, the low sequence diversity and the few diversifying selection signatures observed for *G_07189* could advocate for the durability of quantitative resistance despite a potential gene-for-gene interaction context. In terms of plant breeding, at this stage one can only hypothesize, however the inclusion of what might be called “weak resistance genes” in breeding programs could be a means of generating durable resistance through disease control in the form of favouring “weak” epidemics over the attempt at complete suppression of a given pathogen, thus reducing the selection of highly virulent strains. These lower effect resistance genes could potentially have advantages of both quantitative and qualitative forms of disease resistance. Indeed, although they are *R* genes from a mechanism-based point of view, they induce relatively low selection pressure and thus offer more durable resistance than qualitative *R* genes.

## Data availability statement

The datasets presented in this study can be found in online repositories. The names of the repository/repositories and accession number(s) can be found below: https://www.ncbi.nlm.nih.gov/, PRJNA777581 https://www.ncbi.nlm.nih.gov/, PRJNA881220.

## Author contributions

TM and RV conceived and designed the study. FS obtained the 167 offspring isolates. CL-P and AP performed the cloning and genetic transformations. MC, CBe and SG carried out virulence assays on the offspring isolates, and CL-P on the transformant isolates. CL-P extracted the DNA of the offspring isolates, then CBa and HP ran the RAD-seq experiment. CL-P and NL performed the bioinformatics analyses. AN ran the RT-qPCR experiment. RA performed the population genetic analysis for the candidate effector gene. CL-P and TM analyzed the data and wrote the manuscript. All authors contributed to the article and approved the submitted version.
