# Supplementary material for: Quantitative and qualitative plant-pathogen interactions call upon similar pathogenicity genes with a spectrum of effects
Source: Front Plant Sci. 2023 May 10;14:1128546. doi: 10.3389/fpls.2023.1128546 (PMC10206311; doi:10.3389/fpls.2023.1128546)
Supplement: Supplementary file 1 [file DataSheet_1.docx]

**SUPPLEMENTARY MATERIAL**

**Supporting information 1.** Figure S1
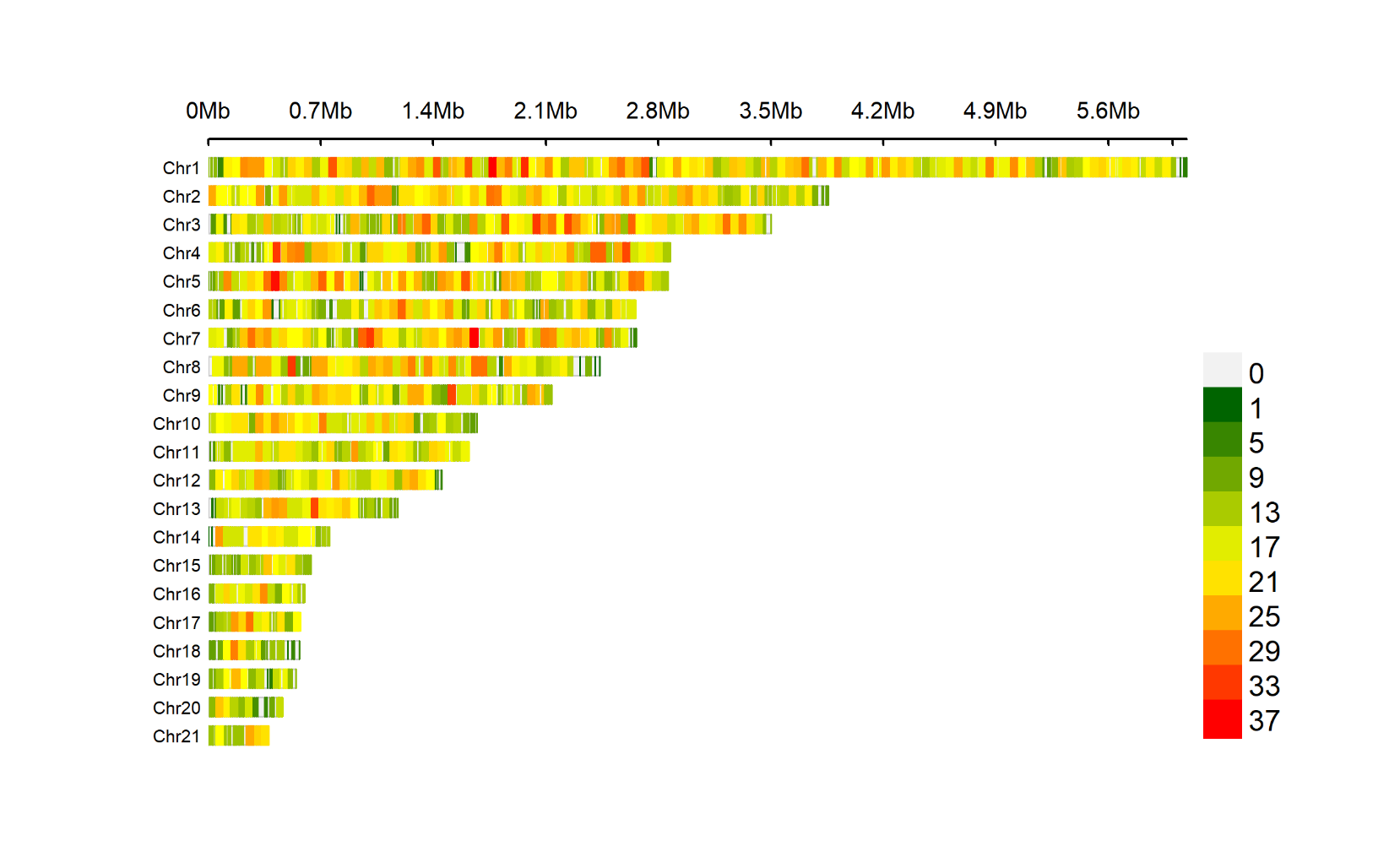


**Figure S1.** Distribution of *Pst1* restriction sites throughout the IPO323 genome. The colour scale indicates the number of restriction sites within a 50kb window. Each line corresponds to a chromosome, 1 through 21.

**Supporting information 2.** Building an Ultra-dense genetic map for *Z. tritici*

*Materials and methods: An ultra-dense genetic map of Z. tritici*

1. SNP identification

The paired-end reads received from the Plateforme MGX were mapped on the reference genome IPO323 with BWAAv0.7.7 using the mem algorithm with default settings (Li and Durbin, 2009). Reads were filtered using samtools v.0.1.19 and picard tools v1.106 (Li and Durbin, 2009; Broad institute, n.d.). All secondary alignments and reads with a mapping quality inferior to 30 were filtered out. SNP identification was carried out with Freebayes V.0.9 with the « --report-monomorphic » option (Garrison and Marth, 2012). All positions detected as low complexity regions or transposable elements (TE) were filtered out. Low complexity regions were detected with RepeatMasker (Smit *et al.*, 2013). TE were detected with REPET (Flutre *et al.*, 2011). For each position, SNP with an allelic frequency inferior to 0.9 were filtered to delete falsely heterozygote SNP, as *Z. tritici* is haploid. A coverage depth filter was applied to delete all SNP with a coverage lower than 5 reads.

1. Construction of an ultra-dense genetic map

SNPs were named according to the chromosome and physical position they mapped to, based on the best BLAST values of the reads on IPO323. The genetic linkage map was built with the Multipoint ultra-dense software (MultiQTL Ltd, Haifa University, Israel). Before the first clustering, all SNPs with over 5% missing data were filtered. Then, a filter to correct any potential segregation distortion was applied, allowing a Chi^2^ up to 9.5. After the first clustering, linkage groups, which belonged to the same chromosome, were merged and the results from Multipoint were transformed with the Kosambi mapping function (Kosambi, 2016).

*Results: An ultra-dense genetic map of Z. tritici*

The genetic data from 167 progeny and both parents led to the detection of 72,899 SNP in total. 89% of SNP were detected in the core-chromosomes and the remaining 11% in the dispensable chromosomes (Table S1). Chromosomes 14 and 18 each carried only 14 detected SNP, they were therefore understood to be absent in the parental strains as can be the case for dispensable chromosomes (Goodwin *et al.*, 2011). These two chromosomes discounted, we obtain an SNP density of 2.1‰ based on IPO-323 chromosome sizes in bp.

**Table S1.** SNP identified in the *Z. tritici* genome using RADseq data

| Chromosome | Number of SNP detected | Number of SNP/ Chromosome size in bp (‰) |
| --- | --- | --- |
| 1 | 11286 | 1.9 |
| 2 | 6544 | 1.7 |
| 3 | 6438 | 1.8 |
| 4 | 4405 | 1.5 |
| 5 | 5346 | 1.9 |
| 6 | 3279 | 1.2 |
| 7 | 5292 | 2 |
| 8 | 4898 | 2 |
| 9 | 4359 | 2 |
| 10 | 3519 | 2.1 |
| 11 | 3563 | 2.2 |
| 12 | 3221 | 2.2 |
| 13 | 2501 | 2.1 |
| 14 | 14 | 0 |
| 15 | 1738 | 2.7 |
| 16 | 1109 | 1.8 |
| 17 | 1837 | 3.1 |
| 18 | 14 | 0 |
| 19 | 1064 | 1.9 |
| 20 | 1385 | 2.9 |
| 21 | 1087 | 2.7 |
| Total without chromosomes 14 and 18 | 72871 | 2.1 |

12 individuals were not taken into account to build the linkage map as they carried missing data for over a cut-off of 10,000 SNP. The map carries 18,316 SNP for 1,332 unique positions distributed in 19 linkage groups each corresponding to a chromosome (Table S2), chromosomes 14 and 18 being absent in the map. The core-chromosomes represent together 89% of mapped markers. The least densely covered core-chromosome is chromosome 6 with 6.8 markers per cM and the most densely covered one is chromosome 7 with 14.9 markers per cM. The distance between consecutive markers ranges from a minimum of 0.95 cM for chromosome 2 to a maximum of 2.39 cM for chromosome 20, the average overall is 1.19 cM. Seven gaps in the map are greater than 10 cM. The three largest are just above 20 cM, on chromosomes 1, 6 and 20. A comparison between the order of the markers on the genetic map and their assumed physical position based on best BLAST values of the reads on IPO323 (Figure S2) shows that overall, the genetic map follows the assumed physical positions of the markers. Chromosome 13 denotes from the rest with an inversed bloc of SNP compared with IPO-323.

**Table S2.** Characteristics of the I05×I07 genetic linkage map

|  | Chromoso-me | Total number of markers | Number of genetic bins | Covered genetic distance (cM) | Marker density (markers/cM) |
| --- | --- | --- | --- | --- | --- |
| Core Chromosomes | 1 | 2878 | 218 | 232.87 | 12.36 |
|  | 2 | 1564 | 125 | 117.94 | 13.26 |
|  | 3 | 1648 | 133 | 136.35 | 12.09 |
|  | 4 | 1170 | 103 | 115.64 | 10.12 |
|  | 5 | 1350 | 107 | 124.2 | 10.87 |
|  | 6 | 743 | 66 | 109.67 | 6.77 |
|  | 7 | 1330 | 71 | 89.19 | 14.91 |
|  | 8 | 1284 | 91 | 93.12 | 13.79 |
|  | 9 | 1126 | 83 | 92.15 | 12.22 |
|  | 10 | 957 | 65 | 82.04 | 11.67 |
|  | 11 | 905 | 63 | 87.95 | 10.29 |
|  | 12 | 788 | 54 | 68.55 | 11.5 |
|  | 13 | 628 | 46 | 63.78 | 9.85 |
| Subtotal 1 | | 16371 | 1225 | 1413.45 | 11.58 |
| Dispensable Chromosomes | 15 | 470 | 26 | 26.3 | 17.87 |
|  | 16 | 213 | 19 | 30.18 | 7.06 |
|  | 17 | 501 | 17 | 18.72 | 26.76 |
|  | 19 | 276 | 22 | 23.93 | 11.53 |
|  | 20 | 231 | 13 | 28.66 | 8.06 |
|  | 21 | 254 | 10 | 17.43 | 14.57 |
| Subtotal 2 | | 1945 | 107 | 145.22 | 13.39 |
| Total | | 18316 | 1332 | 1558.67 | 11.75 |

**
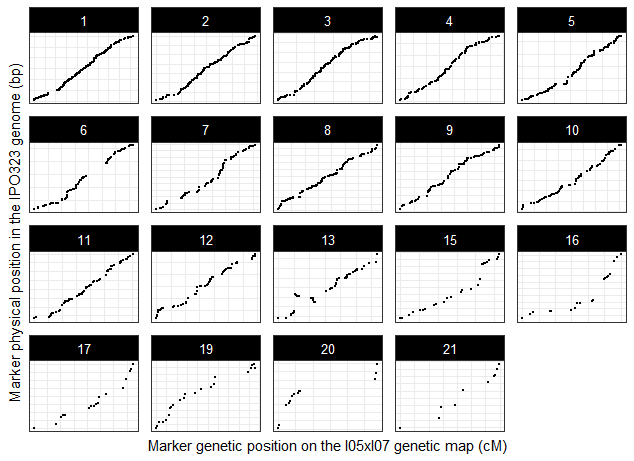
Figure S2.** Comparison between markers’ genetic and assumed physical positions for each chromosome. The X-axis corresponds to the genetic position of the mapped SNP on the I05×I07 genetic map in cM. The Y-axis corresponds to the assumed physical position of the SNP on the chromosome they are on in bp. Each graph represents a chromosome. Each black dot corresponds to a SNP.

*Discussion: An ultra-dense genetic map of Z. tritici*

In this study, we built an ultra-dense genetic map of *Z. tritici* based on RADseq data. It has 18,316 markers for 1,332 genetic positions, called bins. It covers 90% of the genome (chromosomes 14 and 18 discounted) over a genetic distance of 1,559 cM. Lendenmann *et al.* (2014) built two genetic maps using RADseq data. Their maps had 9,745 and 7,333 markers, about half the number in the map presented here. The map presented here therefore offers a far denser coverage than those two previous maps. It does however not cover chromosomes 14 and 18. The previous linkage maps were also subject to presence/absence polymorphisms. Indeed, the 3D1x3D7 map lacked chromosomes 14, 15, 18 and 21, and the 1A5x1E4 map lacked chromosome 17. There are large gaps in the I05×I07 map, the two most problematic of these being those on chromosomes 1 and 6. These is not due to the type of sequencing and lack of *Pst1* restriction sites at certain positions as a closer look into the distribution of restriction sites along the genome debunks this theory, at least in the case of chromosome 1 (Figure S1). It could also be due to complex regions that are difficult to map or simply TE-rich regions, which were filtered out in the SNP calling pipeline. There is a large inversion on chromosome 13. Such inversions could result from a bad assembly of linkage groups, but have also been described as common in fungi and playing an important role in their genome plasticity (Plissonneau *et al.*, 2018). This map led to the identification of three QTL for quantitative pathogenicity with sufficient precision to target particular genes for subsequent work. RADseq is shown to be very well adapted for high-density genetic map building will full-genome coverage. Here from an F1 population we are able to build a map including the quasi-entirety of recombination events, illustrated by the saturation of the map, 18,316 mapped SNP for 1,332 genetic positions.

**Supporting information 3.** I05xI07 population phenotypic data analysis


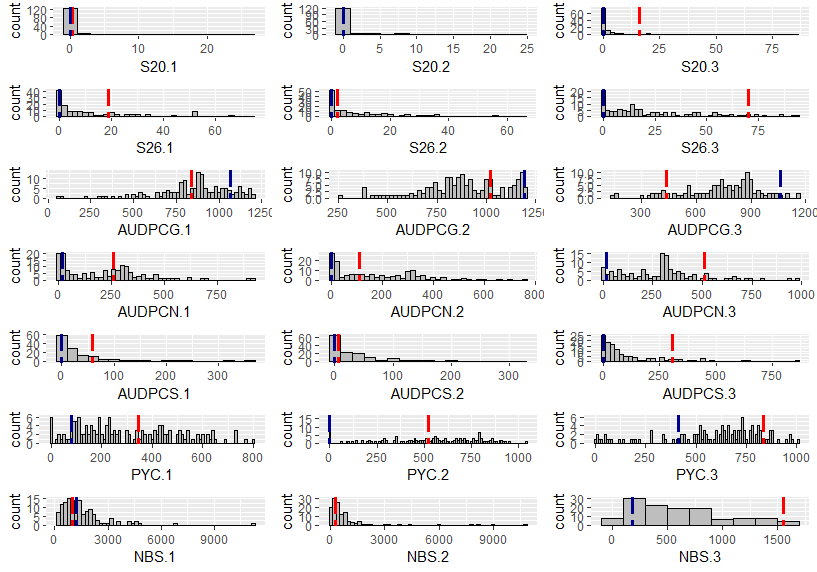


**Figure S3.** Histograms representing the phenotypic data distribution. For all of the histograms, the X-axis represents the trait values and the Y-axis represents the number of individuals from the I05xI07 population, which have the corresponding value for the phenotypic trait. Each line corresponds to a phenotypic trait. S20 and S26 are the sporulating areas (%) at 20 and 26 days respectively and are expressed in percentages. AUDPCs are the Area Under the Disease Progress Curve for the green, necrotic and sporulating areas (AUDPCG, AUDPCN and AUDPCS respectively). PYC is the pycnidia density. NBS is the number of spores per pycnidiospore. Each trait was studied over three replicates: 1, 2 and 3. Each column corresponds to a replicate. The blue dotted line corresponds to the trait value for I07, the red dotted line corresponds to the trait value for I05.

**Table S3.** Statistical analysis of the phenotypic data.

|  | Trait^1^ | Statistical significance of the genotype^2^ | Statistical significance of the replication^2^ | MSg^3^ | MSε^4^ | Broad-sense heritability | Shapiro-Wilk normality test on residuals | Independence of residuals | Homoscedasticity Bartlett test of homogeneity of variances |
| --- | --- | --- | --- | --- | --- | --- | --- | --- | --- |
| No individuals with missing data | S20 | *** | *** | 114.9 | 55.7 | 0.26 | < 2.2E-16 | no | < 2.2E-16 |
|  | S26 | *** | *** | 765 | 160 | 0.56 | 6.23E-06 | yes | 6.893E-04 |
|  | AUDPCG | *** | *** | 91740 | 23116 | 0.50 | 0.06 | yes | 0.4 |
|  | AUDPCN | *** | *** | 74760 | 14626 | 0.58 | 4.24E-03 | yes | 0.11 |
|  | AUDPCS | *** | *** | 20307 | 5800 | 0.45 | < 2.2E-16 | no | 9.78E-12 |
|  | PYC | *** | *** | 79898 | 45477 | 0.20 | 0.1417 | yes | 0.12 |
|  | NBS | ** | *** | 1646264 | 1051014 | 0.16 | < 2.2E-16 | no | < 2.2E-16 |
| only replicates 1 and 2 | S20 | *** |  | 4101 | 1951 | 0.36 | < 2.2E-16 | no | < 2.2E-16 |
|  | S26 | *** | ** | 456.7 | 117.7 | 0.59 | 2.21E-17 | no | 4.71E-03 |
|  | AUDPCG | *** |  | 71061 | 22560 | 0.52 | 0.56 | yes | 0.49 |
|  | AUDPCN | *** | * | 52337 | 12937 | 0.60 | 0.30 | yes | 0.20 |
|  | AUDPCS | *** | * | 8328 | 2356 | 0.56 | 2.37E-12 | no | 6.52E-07 |
|  | PYC | * | *** | 67311 | 47739 | 0.17 | 0.63 | yes | 0.90 |
|  | NBS | * | *** | 2481174 | 1748477 | 0.17 | < 2.2E-16 | no | 3.24E-13 |
|  |  |  |  |  |  |  |  |  |  |
| ^1^ AUDPCs are the Area Under the Disease Progress Curve for the green, necrotic and sporulating areas (AUDPCG, AUDPCN and AUDPCS respectively). S20 and S26 are the sporulating areas (%) at 20 and 26 days post-inoculation respectively. PYC is the pycnidia density. NBS is the number of pycnidiospores per pycnidium.  ^2^ Significance codes: 0 ‘***’, 0.001 ‘**’, 0.01 ‘*’, 0.05 ‘.’, 0.1 ‘ ‘, 1 | | | | | | | | | |
| ^3^ MSg is the mean square value for the individual genotypes output by the ANOVA | | | | | | | | | |
| ^4^ MSε is the mean square value for the residuals output by the ANOVA | | | | | | | | | |

**Supporting information 4.** qPCR on the top candidate gene

*Materials and methods: qPCR on the top candidate gene*

1. Biological material

To obtain *in planta* samples, a pathology assay was carried out according to the same protocol as previously presented. I05 and I07 were inoculated on 'Chinese Spring', 15 pots each containing three plants were inoculated per strain. After inoculation, three samples per strain, a sample here corresponds to all three inoculated leaves in a pot, were collected at 4, 8, 12, 16 and 20 dpi respectively, following a similar kinetic as that used by Gohari *et al.* (2015). Upon collection, all samples were immediately flash-frozen in liquid nitrogen and conserved at -80°C.

In parallel, *in vitro* samples for I05 and I07 were obtained by growing both strains on a PDA (potato dextrose agar) culture medium. Both strains were grown over a week, then subcultured for 4 days before flash-freezing in liquid nitrogen and conservation at -80°C. Three replicates were generated for each strain.

1. RNA extraction

All samples were ground using sterile liquid nitrogen-cooled mortars and pestles. RNA was extracted from each obtained powder using the RNeasy® Mini Kit (Qiagen) according to the supplier’s protocol. Following RNA extraction, a DNase (DNase I, RNase-free (1 U/µL), ThermoFisher Scientific, Waltham, MA, USA) treatment was performed to remove any contaminating genomic DNA.

1. Reverse transcription

cDNA was generated for each sample using ProtoScript® II First Strand cDNA Synthesis Kit (New England Biolabs, Ipswich, MA, USA) using 750ng RNA per reaction.

1. qPCR

Efficiency was determined for all qPCR primers using five-point standard curves generated with genomic DNA, each point as a triplicate (Tables S4 and S5). In the case of the 99-102 primer pair, we cate note that the efficiency is slightly lower than is usually desired at 86.6%, however the other metrics for this primer pair were ideal and the complexity of the region (Figure 5, main text) rendered the design of a more optimal pair impossible. The choice was therefore made to use a primer pair with a very slightly lower efficiency than is conventional.

**Table S4.** Primers used for qPCR

| Number | Name | Sequence 5’-3’ |
| --- | --- | --- |
| 99 | qpcr 6 primer F 1 | GTGCTGTCATCCATTTGTC |
| 101 | qpcr 6 R I05 | GATCGTCCTTGTTCCAAG |
| 102 | qpcr 6 R I07 | TCCCGATCGCTACTGTC |
| 109 | EF1alpha F | AAGATTGGTGGTATCGGAACAG |
| 110 | EF1alpha R | GACTTGACTTCGGTGGTGAC |
| 111 | beta-tubuline F | AACGAGGCTCTCTACGACATCTG |
| 112 | beta-tubuline R | GGCGGAGACGAGGTGGTTG |
| 113 | UBC F | GTCTGCGGACCACAATACC |
| 114 | UBC R | CGACCTTTCCTTGCCTCTG |

**Table S5.** Efficiency of qPCR primer pairs

|  | E (%) | R² |
| --- | --- | --- |
| 111-112 | 97.3 | 0.998 |
| 109-110 | 99.1 | 0.998 |
| 113-114 | 97.1 | 0.998 |
| 99-101 | 94 | 0.996 |
| 99-102 | 86.6 | 0.997 |

qPCR mixes comprised 0.4µL or each primer at 10mM, 5µL of cDNA, 10µL of MESA GREEN qPCR MasterMix Plus for SYBR® Assay (Eurogentec) and 4.2µL of ultra-pure water. Amplification was performed with a 10min step of initial denaturation and enzyme activation at 95°C and 40 cycles of 95°C (10sec) and 60°C (1min). Each sample was run in technical duplicates.

As the primer efficiency was reasonably high (>85%) relative expression was calculated following the 2^-ΔΔCt^ method (Livak and Schmittgen, 2001).

**Supporting information 5.** Molecular cloning detail

*Materials and methods: Molecular cloning detail*

1. Bacterial strains and DNA manipulation

I05 and I07 did not display any resistance to hygromycin and sulfonylurea antibiotics. All primers used are referenced in Table S6. Original vectors pNOV2114 and pNOV_3Gate_SUL were used for the assemblies. Genomic DNA from I05, I07 and IPO323 used to obtain cloning fragment was extracted following the same protocol as for the DNA used for RADseq. For all PCR performed to obtain cloning fragments, the Taq polymerase Phusion (Thermo Fisher Scientific Inc., Waltham, MA, USA) was used under adapted PCR conditions. For the plasmids used to generate knock-out mutants, pNOV2114 was linearized using the primers 15 and 16. A fragment carrying a hygromycin resistance gene and its promoter was obtained by performing a PCR on pNOV_3Gate_HYG using primers 3 and 4. Fragments of approximately 1000bp upstream and downstream of the start and stop of the candidate genes were generated by performing a PCR on genomic DNA of I05, I07 and IPO323 using primers 5 to 10. For the plasmids used to generate complementation mutants and ectopic integration mutants, pNOV_3Gate_SUL, which carries a sulfonylurea resistance gene, was linearized by digestion with KpnI and HindIII following the supplier’s recommendations (Thermo Fisher Scientific Inc., Waltham, MA, USA). Fragments containing the candidate genes and at least 499bp upstream of the start codon and at least 1kb downstream of the stop codon were generated by performing a PCR on genomic DNA of I05 and I07 using primers 22 to 25. The presence of transposable elements with polymorphism between I05 and I07 determined the size of the cloned fragments as we wished to exclude the potential effect of these transposable elements from the experiment. Indeed such regions have been shown to have an effect on pathogenicity (Mat Razali *et al.*, 2019; Seidl and Thomma, 2017; Singh *et al.*, 2021). Assembly of the fragments was performed by Gibson assembly (Gibson *et al.*, 2009; Gibson *et al.*, 2010) using the Gibson Assembly Cloning Kit (New England Biolabs, Ipswich, MA, USA). NEB 5-alpha Competent *Escherichia coli* (High Efficiency) (New England Biolabs, Ipswich, MA, USA) were transformed by heat shock with the generated plasmids and used for their amplification. Successfully transformed strains were identified by PCR and mini-prepped plasmid constructs validated by Sanger sequencing (Eurofins, Luxembourg) using primers 1, 2, 11 to 14, 21, and 26 to 29. The *Agrobacterium tumefaciens* strain AGL1 was then transformed by heat shock with each generated plasmid. Colonies were screened by PCR using the same primers as for the *E. coli* colonies.

- 2. *A. tumefaciens* mediated transformation of *Z. tritici*

The *Z. tritici* strains I05 and I07 were transformed by ATMT (Bowler *et al.*, 2010) following the standard protocol to generate knock-out mutants and ectopic integration mutants. IPO323 was also transformed with the appropriate *A. tumefaciens* strains and used as a transformation control. This enabled us to obtain I05_ΔG07189 and I07_ΔG07189 mutants. I05_ΔG07189 mutant was transformed following the same protocol to generate complementation mutants I05_ΔG07189+G07189_I05_ and I05_ΔG07189+G07189_I07_, and ectopic integration mutant I05+G07189_I07_.

Mutant strains were selected by hygromycin or sulfonylurea screening depending on which resistance gene they were designed to carry. Obtained strains were verified by PCR using primers 17 to 20, 30 and 31 (Table S6) after genomic DNA extraction with DNeasy® Plant Mini Kit (Qiagen) according to the supplier’s protocol.

**Table S6.** Primers used for cloning

| Number | Name | Sequence 5’-3’ |
| --- | --- | --- |
| 1 | pNOV_for | ATGACGCGGGACAAG |
| 2 | pNOV_rev | TAACACATTGCGGATACG |
| 3 | Hygro_for | TGATATTGAAGGAGCAT |
| 4 | Hygro_rev | TCTATTCCTTTGCCC |
| 5 | 5’ QTL6 for | CGGGGATCCTCTAGAGTCGACCATACGCACGATTGCTGTGTTGCTAC |
| 6 | 5’ QTL6 rev | CCAAAAAATGCTCCTTCAATATCAGGTTTTGGGTTGTGGAGATGCACG |
| 7 | 3’ QTL6 for | GTCCGAGGGCAAAGGAATAGATAAAACGAGACGTTCAAATG |
| 8 | 3’ QTL6 rev | TGCGGCCGCTCCGGATTCGAATGCGGTAGCGGAGCTTCTTA |
| 9 | 5’ QTL6 for I07 | gggatcctctagagtcgaccatTGTTAAATACAGCGGTAGTAGAC |
| 10 | 3’ QTL6 rev I07 | CGGCCGCTCCGGATTCGAATAGAGGCTGCCCTTACAACCTCT |
| 11 | Hygro_rev2 | GGGATCAGCAATCGC |
| 12 | Hygro_for2 | CTGCCTGAAACCGAAC |
| 13 | Hygro_rev3 | AGTTGCCTAAATGAACCATC |
| 14 | Hygro_for3 | TCGATGATGCAGCTTG |
| 15 | pNOV_inv_for | ATTCGAATCCGGAGC |
| 16 | pNOV_inv_rev | ATGGTCGACTCTAGAGGATC |
| 17 | Zt-chr6-I07-F1 | ACCCGAATCTACTTTTTGCTGACG |
| 18 | Zt-chr6-I07-R1 | CCCTGCCGGTGGAAGAAGACG |
| 19 | Zt-chr6-I05-F | TCGCATCCGTCATCGCTTCC |
| 20 | Zt-chr6-I05-R | AGGGTCCGTGCATGCTCTTACA |
| 21 | pNOV_sulf_rev | CGCCTGGACGACTAAAC |
| 22 | QTL6 I05 for | CCGAATTCGAGCTCGGTACAGGAGGTCCTGATGGATCGA |
| 23 | QTL6 I05 rev | AATGCTCCTTCAATATCAAAGCTACCGACCCCGGCGG |
| 24 | QTL6 I07 for | AATTCGAGCTCGGTACTCTTGGGCAGGATTCGGATAA |
| 25 | QTL6 I07 rev | ATGCTCCTTCAATATCAACGTAACTAGGCGACGCTAC |
| 26 | seq QTL6 I05 for | CTGTCATCCATTTGTCTGC |
| 27 | seq QTL6 I05 rev | CGTTTTATTACACGCATTCC |
| 28 | seq QTL6 I07 for | CTCTTTCTAATCGGCTAAAACG |
| 29 | seq QTL6 I07 rev | CTCTGCGTCCAAGTATGTG |
| 30 | Pnov_for BIS​ | ​CAGCGGCCATTTAAATC |
| 31 | Sulf_rev​ | ​ATGCTGCCAGTGACACG |

**Supporting information 6.** Table S7

**Table S7.** Details on QTL for pathogenicity detected with the I05×I07 genetic map and phenotypic data

| Replicate | Trait^1^ | 5% threshold (1000 permutations) | Chromo-some | LOD score | Flanking marker 1 | Position flanking marker 1 (cM) | Peak marker | Peak marker position (cM) | Flanking marker 2 | Position flanking marker 2 (cM) | r² (%) | Parent carrying the pathogenic allele |
| --- | --- | --- | --- | --- | --- | --- | --- | --- | --- | --- | --- | --- |
| 1 | AUDPCS | 3.10 | 1 | 4.57 | chr1_244317 | 228.56 | chr1_217589 | 230.58 | chr1_112362 | 232.87 | 7.43 | I05 |
| 1 | S26 | 3.14 | 1 | 5.06 | chr1_244317 | 228.56 | chr1_217589 | 230.58 | chr1_112362 | 232.87 | 7.13 | I05 |
| 2 | AUDPCG | 3.19 | 1 | 3.20 | chr1_250666 | 227.91 | chr1_217589 | 230.58 | chr1_112362 | 232.87 | 5.09 | I05 |
| 2 | AUDPCN | 3.16 | 1 | 3.21 | chr1_244317 | 228.56 | chr1_217589 | 230.58 | chr1_112362 | 232.87 | 4.40 | I05 |
| 2 | AUDPCS | 3.05 | 1 | 3.82 | chr1_244317 | 228.56 | chr1_143600 | 232.54 | chr1_112362 | 232.87 | 6.79 | I05 |
| 2 | S26 | 3.09 | 1 | 4.04 | chr1_244317 | 228.56 | chr1_143600 | 232.54 | chr1_112362 | 232.87 | 6.71 | I05 |
| 3 | AUDPCN | 3.22 | 1 | 4.01 | chr1_244317 | 228.56 | chr1_181231 | 231.90 | chr1_112362 | 232.87 | 6.64 | I05 |
| 3 | AUDPCS | 3.07 | 1 | 3.32 | chr1_244317 | 228.56 | chr1_158919 | 232.22 | chr1_112362 | 232.87 | 6.74 | I05 |
| 1 | AUDPCG | 3.17 | 6 | 7.43 | chr6_366855 | 24.18 | c6.loc30 | 30.00 | chr6_532314 | 34.19 | 20.52 | I05 |
| 1 | AUDPCN | 3.13 | 6 | 11.65 | chr6_366855 | 24.18 | c6.loc28 | 28.00 | chr6_532314 | 34.19 | 30.18 | I05 |
| 1 | AUDPCS | 3.10 | 6 | 8.85 | chr6_366855 | 24.18 | c6.loc28 | 28.00 | chr6_532314 | 34.19 | 23.95 | I05 |
| 1 | PYC | 3.33 | 6 | 16.77 | chr6_366855 | 24.18 | c6.loc28 | 28.00 | chr6_506156 | 31.13 | 40.15 | I05 |
| 1 | S20 | 2.80 | 6 | 3.22 | chr6_363606 | 20.71 | chr6_370491 | 26.54 | chr6_532314 | 34.19 | 9.43 | I05 |
| 1 | S26 | 3.14 | 6 | 10.77 | chr6_366855 | 24.18 | c6.loc28 | 28.00 | chr6_532314 | 34.19 | 28.28 | I05 |
| 2 | AUDPCG | 3.19 | 6 | 7.29 | chr6_366855 | 24.18 | chr6_370491 | 26.54 | chr6_506156 | 31.13 | 20.04 | I05 |
| 2 | AUDPCN | 3.16 | 6 | 10.84 | chr6_366855 | 24.18 | c6.loc28 | 28.00 | chr6_506156 | 31.13 | 28.46 | I05 |
| 2 | AUDPCS | 3.05 | 6 | 6.24 | chr6_366855 | 24.18 | chr6_370491 | 26.54 | chr6_506156 | 31.13 | 17.38 | I05 |
| 2 | S26 | 3.09 | 6 | 7.93 | chr6_366855 | 24.18 | chr6_370491 | 26.54 | chr6_506156 | 31.13 | 21.54 | I05 |
| 3 | AUDPCG | 3.24 | 6 | 8.58 | chr6_370491 | 26.54 | c6.loc28 | 28.00 | chr6_506156 | 31.13 | 29.85 | I05 |
| 3 | AUDPCN | 3.22 | 6 | 11.44 | chr6_370491 | 26.54 | c6.loc28 | 28.00 | chr6_506156 | 31.13 | 37.70 | I05 |
| 3 | AUDPCS | 3.07 | 6 | 6.49 | chr6_366855 | 24.18 | c6.loc28 | 28.00 | chr6_506156 | 31.13 | 23.62 | I05 |
| 3 | NBS | 3.39 | 6 | 3.95 | chr6_363606 | 20.71 | c6.loc26 | 26.00 | chr6_532314 | 34.19 | 13.64 | I05 |
| 3 | PYC | 3.22 | 6 | 11.14 | chr6_370491 | 26.54 | c6.loc32 | 32.00 | chr6_532314 | 34.19 | 33.77 | I05 |
| 3 | S20 | 2.72 | 6 | 3.48 | chr6_363606 | 20.71 | chr6_370491 | 26.54 | chr6_506156 | 31.13 | 12.57 | I05 |
| 3 | S26 | 3.32 | 6 | 9.57 | chr6_366855 | 24.18 | c6.loc28 | 28.00 | chr6_506156 | 31.13 | 32.39 | I05 |
| 2 | S26 | 3.09 | 13 | 3.47 | chr13_1114801 | 0.00 | chr13_1114801 | 0.00 | chr13_978875 | 5.19 | 4.96 | I07 |
| 3 | AUDPCN | 3.22 | 13 | 3.40 | chr13_1114801 | 0.00 | chr13_1114801 | 0.00 | chr13_978875 | 5.19 | 3.22 | I07 |
| 3 | AUDPCS | 3.07 | 13 | 4.64 | chr13_1114801 | 0.00 | chr13_1114801 | 0.00 | chr13_928663 | 8.64 | 7.85 | I07 |
| 3 | NBS | 3.39 | 13 | 4.15 | chr13_1114801 | 0.00 | chr13_1114801 | 0.00 | chr13_978875 | 5.19 | 9.40 | I07 |
| 3 | S20 | 2.72 | 13 | 3.92 | chr13_1114801 | 0.00 | c13.loc4 | 4.00 | chr13_928663 | 8.64 | 8.85 | I07 |
| 3 | S26 | 3.32 | 13 | 5.33 | chr13_1114801 | 0.00 | chr13_1114801 | 0.00 | chr13_978875 | 5.19 | 7.31 | I07 |

^1^ AUDPCs are the Area Under the Disease Progress Curve for the green, necrotic and sporulating areas (AUDPCG, AUDPCN and AUDPCS respectively). S20 and S26 are the sporulating areas (%) at 20 and 26 days post-inoculation, respectively. PYC is the pycnidia density. NBS is the number of pycnidiospores per pycnidium.

**Supporting information 7.** Table S8

**Table S8.** Characteristics of annotated genes within the *Qzt-I05-6* confidence interval

| START (bp) | STOP (bp) | Gene name | Length of coding sequence (bp) | Number of exons | Protein length (aa) | Number of cysteines | Percentage of cysteines | Associated GO terms | Signal peptide |
| --- | --- | --- | --- | --- | --- | --- | --- | --- | --- |
| 366114 | 365404 | Zt09_6_00095 | 621 | 2 | 206 | 3 | 1.46 | 0031505;0005199 | Yes |
| 370562 | 372546 | Zt09_6_00096 | 1677 | 4 | 558 | 7 | 1.25 | 0003824 | No |
| 373148 | 372672 | Zt09_6_00097 | 477 | 1 | 158 | 3 | 1.90 | 0055114;0051537;0016491;0008942 | No |
| 373314 | 375494 | Zt09_6_00098 | 2028 | 3 | 675 | 7 | 1.04 | 0006351;0008270;0003677;0005634 | No |
| 376101 | 377583 | Zt09_6_00099 | 1338 | 3 | 445 | 5 | 1.12 | 0055085 | No |
| 378502 | 377794 | Zt09_6_00100 | 654 | 2 | 217 | 7 | 3.23 | 0016407 | No |
| 379073 | 379997 | Zt09_6_00101 | 759 | 4 | 252 | 3 | 1.19 |  | No |
| 384231 | 380136 | Zt09_6_00102 | 4047 | 2 | 1348 | 2 | 0.15 | 0032065;0005515;0005543;0005938 | No |
| 386490 | 387778 | Zt09_6_00103 | 1206 | 2 | 401 | 5 | 1.25 | 0005515;0005543 | No |
| 389764 | 387944 | Zt09_6_00104 | 1449 | 2 | 482 | 10 | 2.07 | 0016491 | No |
| 390263 | 392435 | Zt09_6_00105 | 1725 | 3 | 574 | 6 | 1.05 |  | No |
| 395620 | 392810 | Zt09_6_00106 | 2811 | 1 | 936 | 13 | 1.39 | 0006298;0005524;0030983;0003677;0032300 | No |
| 397507 | 396649 | Zt09_6_00107 | 795 | 2 | 264 | 5 | 1.89 |  | No |
| 399277 | 397937 | Zt09_6_00108 | 1341 | 1 | 446 | 2 | 0.45 | 0003725 | No |
| 401008 | 399674 | Zt09_6_00109 | 1098 | 4 | 365 | 4 | 1.10 | 0016020 | No |
| 473175 | 471117 | Zt09_6_00110 | 1908 | 3 | 635 | 3 | 0.47 |  | No |
| 473336 | 474422 | Zt09_6_00111 | 900 | 2 | 299 | 0 | 0.00 |  | No |
| 474997 | 477222 | Zt09_6_00112 | 2169 | 2 | 722 | 8 | 1.11 | 0005515 | No |
| 479459 | 477267 | Zt09_6_00113 | 1872 | 5 | 623 | 9 | 1.44 | 0055114;0016614;0050660 | No |
| 480182 | 481778 | Zt09_6_00114 | 1485 | 3 | 494 | 5 | 1.01 |  | No |
| 484136 | 482498 | Zt09_6_00115 | 1581 | 2 | 526 | 7 | 1.33 |  | No |
| 484521 | 486674 | Zt09_6_00116 | 2154 | 1 | 717 | 3 | 0.42 | 0007094 | No |
| 488156 | 487125 | Zt09_6_00117 | 792 | 3 | 263 | 9 | 3.42 |  | No |
| 488690 | 489541 | Zt09_6_00118 | 852 | 1 | 283 | 2 | 0.71 |  | No |
| 490213 | 491217 | Zt09_6_00119 | 1005 | 1 | 334 | 6 | 1.80 |  | No |
| 494292 | 492608 | Zt09_6_00120 | 1575 | 3 | 524 | 5 | 0.95 | 0055114;0004470;0004471;0051287 | No |
| 496252 | 498486 | Zt09_6_00121 | 2235 | 1 | 744 | 2 | 0.27 | 0008289 | No |
| 499583 | 499158 | Zt09_6_00122 | 369 | 2 | 122 | 4 | 3.28 |  | No |
| 501728 | 500457 | Zt09_6_00123 | 1272 | 1 | 423 | 4 | 0.95 | 0071946 | Yes |
| 503651 | 502914 | Zt09_6_00124 | 738 | 1 | 245 | 1 | 0.41 | 0005515 | No |
| 509490 | 511448 | Zt09_6_00125 | 1755 | 4 | 584 | 7 | 1.20 | 0006470;1902751;0004725 | No |
| 520560 | 520022 | Zt09_6_00126 | 399 | 2 | 132 | 1 | 0.76 |  | No |
| 522235 | 523014 | Zt09_6_00127 | 720 | 2 | 239 | 0 | 0.00 |  | No |
| 525247 | 523882 | Zt09_6_00128 | 1245 | 3 | 414 | 2 | 0.48 |  | No |
| 530080 | 531954 | Zt09_6_00129 | 1596 | 5 | 531 | 6 | 1.13 |  | No |
| 532291 | 535332 | Zt09_6_00130 | 3042 | 1 | 1013 | 9 | 0.89 | 0006355;0008134;0003677;0005730 | No |

**Supporting information 8.** Figure S4


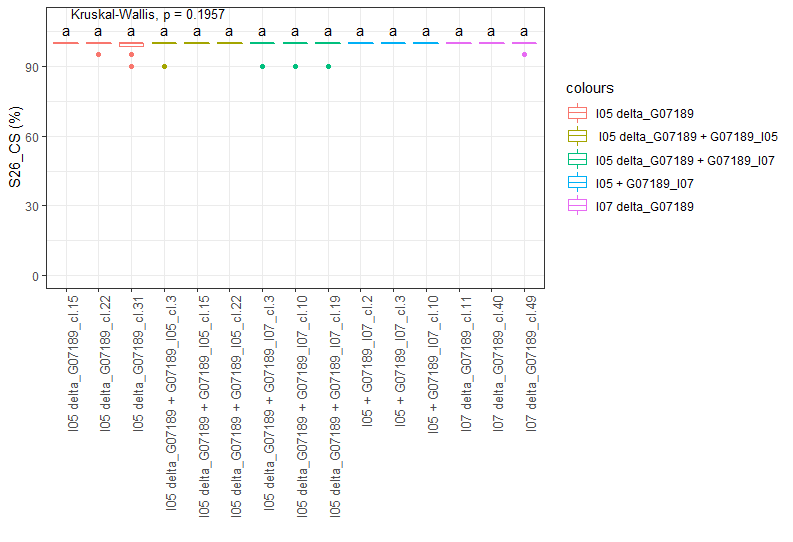

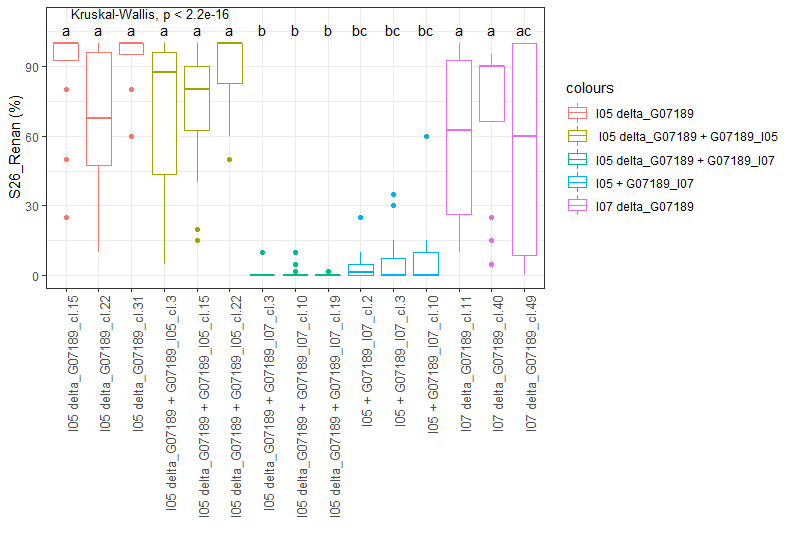


**Figure S4.** Boxplots representing the S26 in % (sporulation a 26 dpi) for all mutant strains generated for *G_07189*. On the left are the results for the inoculation on cultivar 'Chinese Spring', on the right for the inoculation on cultivar 'Renan'. The X-axis represents the trait values for each mutant strain. Colours represent mutant strain types. The Kruskal-Wallis value indicates that the phenotypic value for at least one type of strain is significantly different to the others if p < 0.05. Letters a, b and c indicate significant difference for a Wilcoxon pairwise comparison at alpha=0.05

**Supporting information 9.** Sequence analysis of *G_07189*

**Table S9.** Nucleotide diversity in the *G_07189* sequence.

| Sequence | Nucleotide diversity (π) |
| --- | --- |
| CDS 1 | 0.0001 |
| Intron | 0.00014 |
| CDS 2 | 0.02254 |
| Effector | 0.01152 |

**Table S10.** Model test and parameter estimates of diversifying selection on *G_07189* coding sequence data.

| Model | Parameter estimates | | | lnL | PSS | LRT |
| --- | --- | --- | --- | --- | --- | --- |
| M0: one ratio | ω = 2.118 | | | -498.543 | none | - |
| M1: neutral | p0 = 0.517 | p1 = 0.482 |  | -497.661 | NA | - |
|  | ω0 = 0 | ω1 = 1 |  |  |  |  |
| M2: selection | p0 =0.903 | p1 = 0 | p2 = 0.096 | -487.686 | 3 | M1 vs. M2 (P < 0.001) |
|  | ω0 = 0.772 | ω1 = 1 | ω2 = 21.711 |  |  |  |
|  |  |  |  |  |  |  |
| L; likelihood estimate | | | | | | |
| PSS; number of positively selected sites with p > 0.99 | | | | | | |
| ω; omega dN/dS | | | | | | |
| LRT; likelihood ratio tests | | | | | | |

**References for Supporting information**

Bowler, J., Scott, E., Tailor, R., Scalliet, G., Ray, J. & Csukai, M. (2010) New capabilities for *Mycosphaerella graminicola* research. *Molecular Plant Pathology*, 11, 691–704. https://doi.org/10.1111/j.1364-3703.2010.00629.x

Broad institute Picard Tools - By Broad Institute.

Flutre, T., Duprat, E., Feuillet, C. & Quesneville, H. (2011) Considering transposable element diversification in *de novo* annotation approaches. *PLOS ONE*, 6, 15.

Garrison, E. & Marth, G. (2012) Haplotype-based variant detection from short-read sequencing. *arXiv:1207.3907 [q-bio]*.

Gibson, D.G., Glass, J.I., Lartigue, C., Noskov, V.N., Chuang, R.-Y., Algire, M.A., *et al.* (2010) Creation of a bacterial cell controlled by a chemically synthesized genome. *Science*, 329, 52–56. https://doi.org/10.1126/science.1190719

Gibson, D.G., Young, L., Chuang, R.-Y., Venter, J.C., Hutchison, C.A. & Smith, H.O. (2009) Enzymatic assembly of DNA molecules up to several hundred kilobases. *Nature Methods*, 6, 343–345. https://doi.org/10.1038/nmeth.1318

Gohari, M.A., Ware, S.B., Wittenberg, A.H.J., Mehrabi, R., Ben M’Barek, S., Verstappen, E.C.P., *et al.* (2015) Effector discovery in the fungal wheat pathogen Z*ymoseptoria tritici*. *Molecular Plant Pathology*, 16, 931–945. https://doi.org/10.1111/mpp.12251

Goodwin, S.B., M’Barek, S.B., Dhillon, B., Wittenberg, A.H.J., Crane, C.F., Hane, J.K., *et al.* (2011) Finished genome of the fungal wheat pathogen *Mycosphaerella graminicola* reveals dispensome structure, chromosome plasticity, and stealth pathogenesis. *PLOS Genetics*, 7, e1002070. https://doi.org/10.1371/journal.pgen.1002070

Kosambi, D.D. (2016) The estimation of map distances from recombination values. In: Ramaswamy, R. (Ed.) D.D. Kosambi: Selected Works in Mathematics and Statistics. New Delhi: Springer India, pp. 125–130.

Lendenmann, M.H., Croll, D., Stewart, E.L. & McDonald, B.A. (2014) Quantitative trait locus mapping of melanization in the plant pathogenic fungus *Zymoseptoria tritici*. *G3: Genes, Genomes, Genetics*, 4, 2519–2533. https://doi.org/10.1534/g3.114.015289.

Li, H. & Durbin, R. (2009) Fast and accurate short read alignment with Burrows–Wheeler transform. *Bioinformatics*, 25, 1754–1760. https://doi.org/10.1093/bioinformatics/btp324

Livak, K.J. & Schmittgen, T.D. (2001) Analysis of relative gene expression data using real-time quantitative PCR and the 2−ΔΔCT method. *Methods*, 25, 402–408. https://doi.org/10.1006/meth.2001.1262

Mat Razali, N., Cheah, B.H. & Nadarajah, K. (2019) Transposable elements adaptive role in genome plasticity, pathogenicity and evolution in fungal phytopathogens. *International Journal of Molecular Sciences*, 20, 3597. https://doi.org/10.3390/ijms20143597

Plissonneau, C., Hartmann, F.E. & Croll, D. (2018) Pangenome analyses of the wheat pathogen Zymoseptoria tritici reveal the structural basis of a highly plastic eukaryotic genome. *BMC Biology*, 16, 5. https://doi.org/10.1186/s12915-017-0457-4

Seidl, M.F. & Thomma, B.P.H.J. (2017) Transposable Elements Direct The Coevolution between Plants and Microbes. *Trends in Genetics*, 33, 842–851. https://doi.org/10.1016/j.tig.2017.07.003

Singh, N.K., Badet, T., Abraham, L. & Croll, D. (2021) Rapid sequence evolution driven by transposable elements at a virulence locus in a fungal wheat pathogen. *BMC Genomics*, 22, 393. https://doi.org/10.1186/s12864-021-07691-2

Smit, A., Hubley, R. & Green, P. (2013) RepeatMasker Open-4.0.
